# Spatial Flows of Information Entropy as Indicators of Climate Variability and Extremes

**DOI:** 10.3390/e27111132

**Published:** 2025-10-31

**Authors:** Bernard Twaróg

**Affiliations:** Department of Geoengineering and Water Management, Faculty of Environmental Engineering and Energy, Cracow University of Technology, 31-155 Cracow, Poland; bernard.twarog@pk.edu.pl; Tel.: +48-602129566

**Keywords:** Shannon entropy, entropy flux, source–sink dynamics, climate variability, copula modeling, stochastic modeling

## Abstract

The objective of this study is to analyze spatial entropy flows that reveal the directional dynamics of climate change—patterns that remain obscured in traditional statistical analyses. This approach enables the identification of pathways for “climate information transport”, highlights associations with atmospheric circulation types, and allows for the localization of both sources and “informational voids”—regions where entropy is dissipated. The analytical framework is grounded in a quantitative assessment of long-term climate variability across Europe over the period 1901–2010, utilizing Shannon entropy as a measure of atmospheric system uncertainty and variability. The underlying assumption is that the variability of temperature and precipitation reflects the inherently dynamic character of climate as a nonlinear system prone to fluctuations. The study focuses on calculating entropy estimated within a 70-year moving window for each calendar month, using bivariate distributions of temperature and precipitation modeled with copula functions. Marginal distributions were selected based on the Akaike Information Criterion (AIC). To improve the accuracy of the estimation, a block bootstrap resampling technique was applied, along with numerical integration to compute the Shannon entropy values at each of the 4165 grid points with a spatial resolution of 0.5° × 0.5°. The results indicate that entropy and its derivative are complementary indicators of atmospheric system instability—entropy proving effective in long-term diagnostics, while its derivative provides insight into the short-term forecasting of abrupt changes. A lag analysis and Spearman rank correlation between entropy values and their potential supported the investigation of how circulation variability influences the occurrence of extreme precipitation events. Particularly noteworthy is the temporal derivative of entropy, which revealed strong nonlinear relationships between local dynamic conditions and climatic extremes. A spatial analysis of the information entropy field was also conducted, revealing distinct structures with varying degrees of climatic complexity on a continental scale. This field appears to be clearly structured, reflecting not only the directional patterns of change but also the potential sources of meteorological fluctuations. A field-theory-based spatial classification allows for the identification of transitional regions—areas with heightened susceptibility to shifts in local dynamics—as well as entropy source and sink regions. The study is embedded within the Fokker–Planck formalism, wherein the change in the stochastic distribution characterizes the rate of entropy production. In this context, regions of positive divergence are interpreted as active generators of variability, while sink regions function as stabilizing zones that dampen fluctuations.

## 1. Introduction

Entropy, one of the fundamental concepts in physics, plays a pivotal role in the study of complex climate systems. In its thermodynamic formulation, it describes the degree of disorder and energy dissipation [1,2,3] while in Shannon’s information theory, it serves as a quantitative measure of uncertainty and complexity in data such as temperature and precipitation time series [1,4,5,6]. Contemporary research suggests a significant relationship between physical and informational entropy, opening new avenues for the quantitative assessment of climate variability [7,8].

The Earth’s climate system operates as an open, far-from-equilibrium system in which temperature and pressure gradients drive atmospheric and oceanic circulations [1,9,10]. These processes lead to the transport of heat and moisture and to the production of entropy through energy dissipation, phase transitions, and diffusion [11].

Shannon entropy enables the assessment of uncertainty in the behavior of the climate system, and its analysis across time and space allows for the identification of regions particularly prone to extreme weather events [12,13,14].

In recent years, the concept of information entropy has gained recognition as a powerful tool for characterizing the complexity and unpredictability of climate-related processes. Although explicit references are limited, several hydrological studies—such as those conducted in the Shiyang River Basin—have demonstrated strong conceptual alignment with entropy-based approaches [15].

In particular, the variability of river flows in response to seasonal and spatial fluctuations in precipitation, temperature, and evaporation reflects the uncertainty and dynamic structure that entropy aims to capture. Observed asymmetries in the correlations between streamflow and meteorological factors, along with the effects of permafrost thawing, underscore the need for analytical frameworks that go beyond traditional deterministic measures. Incorporating entropy-based metrics into hydrological modeling can thus enhance our understanding of system instability and climate sensitivity, especially under conditions of increasing variability and anthropogenic change [15,16].

In this study, we analyzed spatial fields and entropy fluxes computed from monthly temperature and precipitation data, employing the formalism of gradients and divergence [1,2,17]. This approach allowed us to capture the directional transport of climate information and to identify local structures of order and chaos that remain undetectable through conventional statistical methods [11,14,18]. The spatial variability of entropy gradients reflects dominant directions of climate variability flow and may indicate external influences such as the impact of the Atlantic Ocean or continentality gradients [19,20,21]. The term “climate variability flow” is not a standard expression and requires clarification. In this study, it refers to the spatio-temporal flow of information related to climate variability, expressed through entropy gradients and fluxes calculated for temperature and precipitation.

Given the use of monthly climatic variables, Shannon entropy was selected due to its balanced sensitivity to distributional variability without amplifying the influence of outliers. While Rényi entropy may provide deeper insight into the behavior of extreme values, it is more suited to high-frequency or event-based data [22]. The spatiotemporal analysis of Shannon entropy enables the identification of areas characterized by elevated distributional uncertainty, which may coincide with regions experiencing heightened climate variability. However, entropy alone does not allow for a quantitative assessment of extremes; to capture rare events more effectively, additional indicators such as kurtosis or alternative entropy formulations (e.g., Rényi entropy) are more appropriate [5,23,24].

The correlation of entropy flux patterns with large-scale atmospheric indices (such as the NAO and AO) enables the linking of local variability patterns with global-scale phenomena [25,26,27]. Regions with elevated entropy relative to their surroundings act as variability generators, whereas areas with low divergence may function as stabilizers of the system, sensitive to external disturbances. Such spatial differentiation enables the identification of entropy sources, sinks, and informational gaps—elements crucial for climate monitoring and forecasting [10,12,28].

The use of Shannon entropy as a measure of uncertainty and complexity in climatic processes was previously proposed in studies such as [29], which demonstrated that entropy effectively captures the dynamics of meteorological systems, particularly in the context of seasonal and spatial variability in precipitation. Building upon this foundation, the present study extends the approach by incorporating both temperature and precipitation into two-dimensional probability distributions, representing a significant methodological advancement.

In the international literature, there is growing interest in the application of copula-based statistical models—such as the Frank, Gumbel, and Clayton copulas—for analyzing the interdependence between climatic variables. Notable examples include studies [30,31], where copulas were primarily used to model the joint occurrence of extreme precipitation and drought events. In contrast, our study employed copula functions in conjunction with entropy estimation, enabling not only the identification of dependencies between temperature and precipitation, but also an information-theoretic assessment of their joint statistical structure—a dimension not addressed in previous research.

Moreover, the application of Mann–Kendall trend tests and Pettitt change-point detection to entropy time series, rather than directly to meteorological variables, represents a less common but promising methodological approach [32]. This enables the detection of subtle shifts in the underlying structure of a non-stationary climate system.

The findings of this study are consistent with those of [33], who demonstrated that spatial entropy increases in regions experiencing intensified seasonal variability—a pattern also observed in our results for Central and Southeastern Europe.

The observed variation in the correlations between entropy measures (including their temporal and spatial derivatives) and indicators of extreme weather events is consistent with the findings of Aristov et al. (2022) [34], who demonstrated that data resolution, local topographic conditions, and climate type significantly modulate the statistical relationship between entropy and meteorological parameters.

In summary, this study not only validates previous findings, but also introduces an integrated framework that combines copula analysis, time-series evaluation, and spatial entropy mapping. The proposed methodology offers a complementary perspective to traditional analyses of climatic trends and extremes, demonstrating strong consistency with existing regional and global studies, while also contributing novel insights into the multivariate characterization of climate variability.

The method of analyzing spatial entropy flows proposed in this study represents an innovative approach to investigating climate variability, going beyond the capabilities of classical statistical tools [35]. Unlike conventional trend and anomaly analyses based on simple descriptive statistics—such as means, standard deviations, or the frequency of extremes—the information-theoretic tools applied here enable the detection of subtle structural changes in the distribution of climatic data.

A particularly novel aspect is the use of spatiotemporal derivatives of Shannon entropy to identify local directions of climatic information flow, as well as their relationships with extreme weather events [36,37]. By employing the formalism of gradients and field theory, this analysis allows for the detection of regions that generate atmospheric instability (entropy sources) and zones that act to stabilize the system (entropy sinks). Through integration with the Fokker–Planck framework, the method also enables the interpretation of observed changes as the result of the drift and diffusion of information in a stochastic system.

Another crucial element is the use of copulas to model asymmetric dependencies between climatic variables, allowing for a more accurate representation of their interrelationships, particularly in the distribution tails [38,39,40]. Additionally, the application of the block bootstrap method provides estimates of uncertainty and statistical significance, thereby enhancing the reliability of the results [41,42,43,44].

In contrast to classical linear regression, the method presented here does not assume linearity or stationarity, making it better suited to the nonlinear and transition-prone nature of atmospheric systems. The spatial perspective on entropy also enables the mapping of informational gaps—regions where modeling is challenged by a lack of structured patterns. This type of analysis not only helps identify areas of elevated risk, but also yields insights into the underlying structure of the climate system itself.

Particularly valuable is the method’s ability to capture regime transitions in atmospheric circulation, as demonstrated by the observed associations between entropy gradients and large-scale indices such as NAO and GISTEMP [37,45]. Thus, the proposed method offers not only a quantitative, but also a qualitative perspective on climate variability, serving as a bridge between classical climatology and modern complex systems analysis. As a result, it supports the formulation of more robust hypotheses regarding the origin of extreme atmospheric phenomena.

Through spatiotemporal entropy analysis, it also becomes possible to gain a deeper understanding of the processes driving local climate fluctuations. From a practical standpoint, this methodology may serve as a decision-support tool for climate adaptation planning, thereby making a significant contribution to the development of advanced tools for assessing climate system dynamics [40,46].

## 2. Data Preparation for Analysis

The analysis presented in this study was based on high-quality gridded datasets published by the National Oceanic and Atmospheric Administration (NOAA [47,48,49]): Terrestrial Precipitation: 1900–2010 Gridded Monthly Time Series (Version 3.01) and Terrestrial Air Temperature: 1900–2010 Gridded Monthly Time Series (Version 3.01). The study area covers approximately 5570 km × 4050 km, spanning latitudes from 10° S to 40° N and longitudes from 35° E to 72° E.

The analysis used monthly precipitation totals and monthly mean air temperatures for the period 1901–2010 [47,50]. The data were provided by NOAA as part of a climate reanalysis product and interpolated onto a regular angular grid with a spatial resolution of 0.5° × 0.5°, centered at 0.25°.

For each grid cell, individual time series of temperature and precipitation were extracted and served as the basis for subsequent analyses grounded in informational entropy.

The NOAA data were utilized at their original resolution, without re-interpolation or grid rescaling. A major reason for selecting this dataset was its temporal and spatial homogeneity. Both temperature and precipitation fields were developed using standardized interpolation algorithms and quality control procedures, applied consistently by the same institution. This ensures a high level of data integrity and facilitates comparability across regions and time periods.

To ensure the reliability of long-term trend analyses, NOAA datasets are routinely verified and homogenized. This includes statistical identification of anomalies, data gaps, and inconsistencies, as well as cross-validation with ground-based observations and satellite products [48,49,51]. As a result, the NOAA dataset provides a stable and trustworthy foundation for detecting climate variability signals associated with entropy flow, as well as for analyzing the spatial directions of climate information transport.

In the conducted comparative analysis of meteorological data, the consistency and reliability of monthly NOAA reanalysis data were assessed through comparison with an independent observational dataset—E-OBS, developed by the Copernicus Climate Change Service [51,52]. The E-OBS data, available at a daily resolution of 0.25° × 0.25°, were first aggregated into monthly precipitation totals and mean temperatures for the period 1980–2010. In the next step, bilinear spatial interpolation was applied to align E-OBS data with the NOAA grid, thereby enabling direct, point-by-point comparisons with the gridded NOAA dataset. Only after ensuring temporal and spatial consistency were grid-cell-level comparisons carried out. This careful data preparation allowed for a robust assessment of the agreement between the two datasets in terms of monthly distributions, long-term trends, and the temporal structure of variability.

The validation results indicated strong agreement between the datasets. For precipitation, the relative root mean square error (RRMSE) rarely exceeded 0.1, indicating minimal discrepancies relative to E-OBS [53,54]. Mean monthly differences between the precipitation datasets were below 5 mm/month in most areas, and high Spearman rank correlations (>0.8) confirmed a strong match in the temporal variability structure. Even greater consistency was observed in the temperature data: RRMSE values were below 0.05 across much of the grid, the average temperature difference between NOAA and E-OBS was less than 2 °C in 95% of cases, and rank correlations exceeded 0.95 at most locations [55]. Observed local discrepancies—primarily in mountainous or coastal regions—can be attributed to interpolation limitations and local characteristics of the observational network.

To ensure data quality, all NOAA time series were screened for missing data, and grid points with incomplete temporal coverage (<360 months) were excluded from further comparison. No imputation of missing data was applied, in order to avoid introducing systematic bias. This approach ensured fair comparative conditions and high confidence in the conclusions.

In light of the analysis, it can be stated with high confidence that the NOAA reanalysis data showed very strong agreement with the E-OBS observational dataset and can be considered a reliable source for further studies on climate variability, both in the temporal and spatial dimensions.

## 3. Methodology

This study investigated the relationships between the structure of informational entropy and extreme weather phenomena, with a particular focus on maximum temperatures and minimum precipitation totals [56,57].

This approach is based on the analysis of annual values of entropy and their spatial gradients (entropy potential) for individual months, calculated at grid points covering the European domain. For each grid point, monthly values of information entropy were computed based on the joint distributions of atmospheric conditions—temperature and precipitation.

Although the input data had a monthly resolution (monthly mean temperatures and precipitation totals), entropy was not calculated based on individual monthly observations. Instead, a moving time-window approach was employed: for each grid point and each calendar month (e.g., January, February, etc.), a sample of 70 consecutive years was extracted. Within each window, the joint distributions of temperature and precipitation were estimated, and the corresponding entropy values were calculated. This process was repeated by shifting the window one year forward (e.g., 1901–1970, 1902–1971, and so on).

In this way, a time series of entropy values was obtained for each calendar month, where each value represents 70-year conditional statistics, rather than single monthly observations. For simplicity, we refer to these values throughout the manuscript as entropy estimated in a 70-year window for a given calendar month. For example, the entropy for January 1971 was based on data from 1901 to 1970, for January 1972 on data from 1902 to 1971, and so forth. As a result, we obtained 40 seasonal annual entropy values (calculated within a 70-year window for each calendar month) to which a time index can be assigned, enabling trend estimation and other temporal analyses.

For each grid point, entropy was computed separately for each calendar month, based on the joint distributions of atmospheric conditions (temperature and precipitation). To capture the actual, often nonlinear and asymmetric dependence structure between temperature and precipitation in a changing climate system, bivariate copula functions were used [20,58].

The approach is based on the analysis of monthly entropy values and their spatial gradients (entropy potential) across a grid covering the European domain [2,12,26,59]. 

Additionally, for each month, the spatial gradient of entropy was computed and interpreted as a vectorial entropy potential, with its magnitude (|∇Entropy|) serving as a proxy for local atmospheric variability and instability. The direction and rate of change in uncertainty across space were defined by the entropy gradient vector (∇Entropy) [60,61].

The primary objective of the analysis was to determine whether statistically significant relationships existed between entropy levels and their spatial dynamics, on the one hand, and extreme values of maximum/minimum temperature and precipitation, on the other hand, at the same geographic locations. Three types of relationships were examined: (1) between the entropy value (Entropy) and weather extremes, (2) between the entropy potential (|∇Entropy|) and extremes, and (3) between the temporal derivative of entropy and extremes [57,60].

The first approach tested whether higher uncertainty levels (greater entropy) at a given location correspond with increased risk of high temperatures or drought. A positive correlation between entropy and maximum temperature would suggest that regions with higher variability are more prone to experiencing extreme heat events. Conversely, a negative correlation between entropy and minimum precipitation totals would imply that higher uncertainty favors the occurrence of severe droughts [36,62,63].

The second approach focused on the analysis of the entropy gradient, interpreted as the rate and direction of spatial entropy changes. The magnitude of the gradient vector (|∇Entropy|), or entropy potential, was used to identify regions of pronounced spatial instability that may serve as initiation zones for extreme events. The analysis thus examined whether sharp spatial variations in entropy are linked to the occurrence of extreme temperature and precipitation values [60,64,65].

All correlations were computed using two variants: temporal and seasonal–spatial. In the temporal analysis, for each grid point, the Spearman correlation was calculated between the 12-month entropy or entropy gradient series and the corresponding monthly values of weather extremes [66]. The resulting correlation coefficient indicated the strength and direction of the relationship between monthly entropy fluctuations and variations in extreme weather events.

While Spearman’s rank correlation is a useful tool for general data exploration, it is not optimally suited for characterizing relationships between extreme events. In particular, for variables with highly asymmetric distributions or heavy tails—such as extreme precipitation or temperature maxima—classical correlation measures may fail to accurately capture the underlying dependence structure [46,67]. In such cases, more appropriate approaches involve tail dependence analysis including the use of copulas, tail indices, or conditional exceedance probabilities. Therefore, the current approach should be viewed as a preliminary step in identifying potential dependencies, rather than a definitive method for their validation

A high positive temporal correlation signified that months with elevated entropy potential coincided with months of extreme conditions (e.g., heatwaves), while a high negative correlation indicated an inverse relationship—i.e., that increased local instability was associated with lower extreme values, such as droughts. In the seasonal–spatial variant, spatial correlations were examined within each month separately. Spearman correlations were computed between the spatial distribution of entropy (or entropy gradients) and the spatial distribution of weather extremes for a given month [53,66]. This analysis addressed whether, during a given season, areas with higher entropy potential also exhibited a greater risk of extreme weather. These spatial correlations provided insight into the general seasonal–spatial structure of the relationship between climate variability and extremes [68].

To enhance the reliability of the results, we applied a circular block bootstrap resampling procedure, which preserves the autocorrelation structure typical of time series constructed from overlapping 70-year windows. Instead of simple resampling of individual observations with replacement, entire blocks of adjacent values were drawn. The block length was determined individually based on the analysis of the autocorrelation function (ACF) of the residuals, after detrending the data—specifically, using the minimum lag at which the ACF ceased to be statistically significant.

For each time series, 1000 bootstrap replications were generated, allowing us to obtain empirical distributions of the trend slope estimates as well as reliable confidence intervals. This procedure increases the robustness of the results to Type I errors, which may arise when autocorrelation is ignored in classical statistical tests.

Particular attention was given to the Spearman rank correlation coefficient, whose values were interpreted at the 5% significance level (corresponding to a 95% confidence interval) [12,69,70]. This approach allowed not only for the estimation of the average strength of dependence, but also for the assessment of the stability of the results under the strong autocorrelation conditions typical of the analyzed time series.

The results were presented as spatial maps, including distributions of entropy, trends in entropy and entropy potential, temporal and seasonal–spatial correlation maps, and visualizations of entropy gradient streamlines. The streamlines indicated trajectories along which uncertainty propagates spatially—offering potential prognostic value. Particularly promising were the findings from the entropy gradient analysis, which pointed to the existence of spatial mechanisms that may initiate extreme weather events. Strong local entropy gradients may signal that the atmospheric system is approaching bifurcation thresholds that lead to more chaotic states.

Strong local entropy gradients may be interpreted as a potential signal that the atmospheric system is approaching bifurcation thresholds—an indication which, according to dynamical systems theory (e.g., Scheffer et al.) [71], is often associated with increased susceptibility to transitions toward more chaotic states. This interpretation is hypothetical in nature and requires further empirical validation.

### 3.1. Distribution Fitting

The methodology presented in this study was based on bivariate copula functions, which offer a coherent and statistically well-founded approach to modeling dependencies between random variables with arbitrary marginal distributions. This approach allows for the separation of marginal modeling from dependence structure modeling, enhancing both flexibility and interpretability of the results.

The analytical process began with the estimation of marginal distribution parameters using the maximum likelihood estimation (MLE) method. This method provides efficient and consistent estimators, assuming standard regularity conditions are met. For each fitted marginal distribution, the Akaike Information Criterion (AIC) was applied to objectively select the best-fitting model, balancing model complexity and goodness of fit [6,72,73].

The selected marginal distributions were then transformed into the uniform space using their corresponding cumulative distribution functions (CDFs), which is a standard step in copula construction. In the next stage, the parameters of the selected copula functions (e.g., Clayton, Gumbel, Frank, Gaussian, and Student-t copulas) were also estimated using MLE [43,55,74]. This step enabled the full dependence structure to be captured independently of the marginal distributions.

Among the fitted copula functions, the one that best captured the dependencies observed in the data was selected. In addition to the Akaike Information Criterion (AIC), the primary selection criterion was the agreement between the empirical joint distribution function and the theoretical distribution derived from the copula model [27,45].

This ensures that the entire process is grounded in comparable and statistically robust measures of goodness-of-fit, eliminating arbitrariness in the choice of both margins and dependency structures. Such a methodology adheres to the standards of modern probabilistic modeling, integrating marginal and joint characteristics, and proves particularly useful in meteorological, hydrological, and financial applications.

Importantly, given a sufficiently large sample size and careful selection of candidate marginal and copula functions, this procedure enables not only the accurate description of dependencies, but also the prediction of extreme events and the assessment of joint risks associated with their co-occurrence. The framework thus constitutes a coherent and calibratable approach for constructing bivariate probabilistic models with strong statistical foundations.

#### 3.1.1. Marginal Distributions

The modeling of Shannon entropy in this study relies on temperature and precipitation data. For each spatial grid cell, marginal distributions were analyzed separately for monthly mean temperatures and monthly total precipitation values.

The following distributions were considered as candidates for marginal fitting (see Table 1) [55]: Generalized Extreme Value (GEV), Normal, Log-normal, Weibull, Gamma, Extreme Value, Nakagami.

Each distribution was fitted using the maximum likelihood estimation (MLE) method, and its goodness of fit was assessed using the Anderson–Darling test. The optimal model was selected based on the Akaike Information Criterion (AIC), which allowed for consideration of local climatic conditions and regional differences in the variability of temperature and precipitation across Europe.

#### 3.1.2. Bivariate Copula Functions

The application of bivariate copula functions in analyzing the joint variability of temperature and precipitation enabled a precise representation of the dependencies between these variables, particularly in the context of Shannon information entropy estimation. Traditional approaches that assume independence or simple linear correlation are insufficient for capturing the true, often nonlinear and asymmetric dependence structure that characterizes the relationship between temperature and precipitation in a variable climate system. Copulas, as statistical tools, allow for the decoupling of marginal distributions from the dependence structure, making it possible to model interactions even in the presence of strong asymmetries, tail dependencies, or extreme values [75,76].

This study employed four classic families of copulas: Gaussian, Clayton, Frank, and Gumbel, each contributing distinct interpretative strengths to climate analysis (see Table 2) [77,78,79].

The Gaussian copula, an extension of the multivariate normal distribution, is effective in capturing symmetric dependencies, but it does not account for tail dependence, which limits its utility in analyzing extreme co-occurrences such as droughts or heatwaves. Nevertheless, it remains valuable where dependence is moderate and variables do not deviate strongly from normality [75].

The Clayton copula, characterized by strong lower-tail dependence, is well-suited for analyzing the co-occurrence of low precipitation and high temperatures, conditions typical of droughts. This makes it especially useful in exploring regional seasonal drought patterns, where standard methods fail to capture intense dependence under extreme conditions [45].

In contrast, the Gumbel copula emphasizes upper-tail dependence, making it appropriate for modeling extreme co-occurring events, such as intense rainfall during heat-induced convective storms, where high temperatures promote evaporation and condensation [80,81].

The Frank copula offers greater flexibility in modeling moderate and symmetric dependencies, without favoring either tail. It is therefore used as an intermediate model, particularly where dependencies are evident but not extreme [81].

The Akaike Information Criterion (AIC) was used to select the best-fitting copula at each grid point, allowing for localized adaptation of the model to regional climatic conditions. This enabled a faithful reconstruction of the complex landscape of temperature–precipitation dependencies across the continental scale. A key advantage of this approach lies in its ability to accurately estimate the joint distribution of variables, which then serves as the basis for computing Shannon entropy [46,76,79,82].

The spatial analysis of entropy derived from copula-based distributions allows for the identification of regions with distinct dependency structures (e.g., transition zones, areas of increased continentality, or regions influenced by orographic barriers). Furthermore, spatial differentiation in copula types reveals where particular extremes dominate—such as droughts or heavy rainfall—which has critical implications for climate risk assessment [46].

Integrating copula functions with information entropy analysis represents a significant step toward a more comprehensive description of complex climate interactions. This approach combines the probabilistic rigor of dependence modeling with the interpretive strength of information theory. Since entropy estimated from copulas captures the entire dependence structure, rather than just correlation, it allows for the inclusion of subtle, locally specific climatic features. This proves especially effective in monthly data analysis, where distributions often vary seasonally and regionally, and traditional multivariate models fall short in reflecting the full complexity of dependencies. Ultimately, copula functions not only improve the accuracy of entropy estimation, but also enable inference about the causes and nature of extreme weather events, supporting the development of more advanced tools for monitoring and forecasting climate variability.

In this study, a semi-parametric approach was adopted which—while ensuring mathematical consistency and high operational efficiency—also involves a certain methodological trade-off. The use of established families of distributions and copulas allows for the straightforward derivation of derivatives, efficient computation of entropy, and implementation of estimation algorithms. At the same time, adopting a parametric model inevitably entails some simplification of the data structure: local features of the empirical distribution, such as asymmetry, multimodality, or unusual tails, may be partially smoothed or overlooked. This is the cost of increased interpretability and analytical tractability of the model, particularly in the context of entropy gradient analysis or modeling of information fluxes.

An alternative would be a hybrid approach, combining parametric marginal distributions with nonparametric copulas (e.g., kernel-based), or a fully nonparametric variant based on empirical distribution functions and flexible adaptive or rotational copulas. Although potentially more accurate, such methods involve substantially higher computational costs, reduced numerical stability, and more challenging interpretation. Given the aim of this study—comprehensive spatio-temporal analysis of information entropy—the use of parametric models represented a justified compromise between estimation accuracy and analytical capability.

Although it is theoretically possible to estimate the joint entropy H(T,P) empirically from 70 observation pairs within a moving window (e.g., using k−NN estimators or via discretization with the Miller–Madow correction), we deemed this approach unsuitable in our case [83]. With such a limited sample size, two-dimensional differential entropy estimators operate at the edge of stability, are highly sensitive to technical parameter choices, and produce large variance, which hampers comparability across locations. In addition, the strong autocorrelation present in climate series within 70-year windows means that even bootstrap procedures yield wide, weakly informative confidence intervals. As a result, empirical entropy estimates would carry substantial uncertainty and be difficult to interpret. For these reasons, we opted for the semi-parametric approach, which ensures greater consistency and reproducibility of results across the entire analysis.

For the analysis of the bivariate distributions of temperature and precipitation, we employed a semi-parametric approach based on selecting marginal distributions and copulas from a limited set of candidate functions. For each marginal variable, seven univariate distributions were considered, while the bivariate dependencies were modeled using one of four copulas: Frank, Gaussian, Clayton, or Gumbel. Model selection for the marginal distributions and copulas was carried out separately for each grid point and calendar month, based on the full 110-year data series (1901–2010). The selected model was then kept fixed throughout the time series, with only its parameters updated within successive 70-year windows.

This approach has two key advantages:

- it ensures consistency and comparability of results over time at each grid point, by avoiding artificial distortions in entropy analysis that could arise from changing model selections;
- it reduces the risk of overfitting by preventing arbitrary model adaptation to short samples.

The limitation of this method is the potential for the imperfect fit of a single fixed model across the entire observation period, particularly if the structure of climate dependencies changes over time. Nevertheless, in the context of trend and entropy dynamics analysis, the benefits of modeling consistency and stability were judged to outweigh this limitation, and any potential misfit is expected to be systematic in nature, without distorting the relative changes over time.

#### 3.1.3. Akaike Information Criterion (AIC)

The marginal distributions describing temperature and precipitation for each analyzed sequence were estimated using the maximum likelihood method, assessed via the Anderson–Darling test (ADT). The selection of the optimal model was based on Akaike Information Criterion (AIC) values and the goodness-of-fit between empirical and theoretical distribution functions.

AIC is a model selection metric that balances goodness-of-fit with model complexity. It is defined as:

(12) $$AIC=2k-2ln({\hat{L}}_{max})$$

where: k is the number of estimated model parameters and ${\hat{L}}_{max}$ is the maximum likelihood value of the model.

A lower AIC value indicates a better trade-off between fit and simplicity.

The log-likelihood function used in the computation is given by:

(13) $$\ln\left(\hat{L}\left(\theta \left|x_{1},x_{2},\ldots ,x_{n}\right.\right)\right)=\sum_{i=1}^{n}lnf\left(x_{1}\left|\theta \right.\right)$$

where $f\left(x_{1}\left|\theta \right.\right)$ is the probability density function of the model evaluated at observation $x_{1}$ given parameter vector θ.

#### 3.1.4. Anderson–Darling Test (AD Test)

The Anderson–Darling test is particularly useful in the analysis of climatic and hydrological variables as it provides a reliable assessment of distributional fit even in the extremes of the distribution, which is crucial for studies involving precipitation and temperature.

It belongs to the family of goodness-of-fit tests and represents an extension of the classical Kolmogorov–Smirnov test. The core idea is to compare the empirical cumulative distribution function $F_{n}(x)$ with a theoretical cumulative distribution function F(x) of the candidate distribution. In contrast to the KS test, the Anderson–Darling test assigns greater weight to discrepancies in the tails of the distribution.

The test statistic is defined as:

(14) $$A^{2}=-n-\frac{1}{n}\sum_{i=1}^{n}\left[\left(2i-1)(lnF\left(X_{i}\right)+ln(1-F\left(X_{n+1-i}\right))\right)\right]$$

where $X_{i}$ denotes the ordered sample values and n is the sample size.

Large values of the $A^{2}$ statistic indicate a lack of fit between the sample and the theoretical distribution.

### 3.2. Statistical Tests Used

To assess trends in Shannon entropy for both precipitation and temperature, the block bootstrap resampling technique was employed to generate multiple statistical realizations and to estimate stable entropy values. For each iteration, a separate estimation of the marginal distribution parameters was performed, followed by the construction of a joint distribution using copula functions.

#### 3.2.1. Pettitt Test (PCPT)

The PCPT has been widely used to detect changes in observed climatic and hydrological time series [8,16]. The Pettitt test is also applicable to investigate an unknown change point by considering a sequence of random variables ${(X}_{1},\;X_{2},...,\;X_{T})$, which have a change point at τ. As a result, $\left(X_{1},\;X_{2},...,\;X_{\tau }\right)$ has a common distribution $F_{1}(\cdot )$ but $\left(X_{\tau +1},\;X_{\tau +2},...,\;X_{T}\right)$ has a different distribution $F_{2}(\cdot )$, where $F_{1}(\cdot )\neq F_{2}(\cdot )$. The null hypothesis $H_{0}$ (no change but τ=T) was tested against the alternative hypothesis $H_{1}$ (change 1≤ τ <T) using the non-parametric statistic $K_{T}\;=\;max|U_{t,T}|=max\;(K_{T+}\;,\;K_{T-})$ where:

(15) $$U_{t,T}=\sum_{i=1}^{t}\sum_{j=t+1}^{T}sgn(X_{t}-X_{j}),$$

(16) $$sgn\left(X_{t}-X_{j}\right)=\left[\begin{array}{cc} 1 & (X_{t}-X_{j})>0\\ 0 & (X_{t}-X_{j})=0\\ -1 & (X_{t}-X_{j})<0 \end{array}\right],$$

$K_{T+}\;=maxU_{t,T}$ for the downward shift and $K_{T-}\;=\;-minU_{t,T}$ or the upward shift. The confidence level associated with $K_{T+}$ lub $K_{T-}$ is approximately determined by:

(17) $$\rho =exp(\frac{-6{K_{T}}^{2}}{T^{3}+T^{2}}),$$

When  p is smaller than the specified confidence level (for example, in this study, 0.95 was adopted), the null hypothesis is rejected. 

The approximate p-value for the change point is defined as:

(18) p=1−ρ,

In this study, the Pettitt test was specifically applied to identify change points in the time series of Shannon entropy derived from monthly precipitation totals and average monthly temperatures. PCPT, based on a test statistic compared against a critical value, allows for determining whether the null hypothesis of no abrupt change can be rejected [84]. This method is widely used in climatological and hydrological analyses due to its robustness in detecting structural changes in environmental time series [12]. The test was applied recursively: after identifying the first change point at the 5% significance level, the corresponding segment was removed and the remaining data were analyzed again.

#### 3.2.2. Modified Mann–Kendall Trend Test (MMKT)

Trend characteristics and patterns in entropy were analyzed using the Modified Mann–Kendall Test (MMKT)—a nonparametric statistical test widely used in studies of climate change [85,86]. Null hypotheses were rejected at a significance level of α = 0.05, corresponding to a 95% confidence level. The choice of this method was motivated by the fact that climate time series based on 70-year moving windows exhibit strong autocorrelation, which in the classical Mann–Kendall test leads to underestimation of the variance of the S statistic and thus the overstatement of trend significance.

The solution proposed by Hamed and Rao [85] introduces a correction to the variance:

(19) $${Var}^{*}\left(S\right)=Var(S)\cdot n^{*}$$

where $n^{*}$ is the effective sample size, computed from the autocorrelation function $\rho_{k}$ of the residuals at lag k, after removing the trend:

(20) $$n^{*}=\frac{n}{1+\frac{2}{n(n-1)(n-2)(n-3)}\sum_{k=1}^{n-1}\left(n-k\right)\left(n-k-1\right)(n-k-2)\rho_{k}}$$

The corrected test statistic is given by:

(21) $$Z^{*}=\left\{\begin{array}{cc} \frac{S-1}{\sqrt{{Var}^{*}\left(S\right)}} & S>0\\ 0 & S=0\\ \frac{S+1}{\sqrt{{Var}^{*}\left(S\right)}} & S<0 \end{array}\right\},$$

where the S statistic is calculated as:

(22) $$S=\sum_{i=1}^{n-1}\sum_{j=i+1}^{n}sgn(x_{j}-x_{i}),$$

and:

(23) $$sgn\left(x_{j}-x_{i}\right)=\left[\begin{array}{cc} 1 & (x_{j}-x_{i})>0\\ 0 & (x_{j}-x_{i})=0\\ -1 & (x_{j}-x_{i})<0 \end{array}\right],$$

The null hypothesis of no trend is rejected when $\left|Z^{*}\right|>Z_{1-\frac{\alpha }{2}}$ (i.e., 1.96 for α = 0.05).

In addition, to identify change points, the Pettitt test was applied [66,69,84]. When a statistically significant change (α = 0.05) was detected, the time series was divided into subsequences, each of which was then re-analyzed using the Modified Mann–Kendall Test (MMKT). If no change point was identified, MMKT was applied to the entire series. This approach enabled the simultaneous detection of monotonic trends and potential regime shifts in the data.

#### 3.2.3. Hirsch–Sen’s Slope Estimator

To quantitatively assess the magnitude of the trend, a non-parametric slope estimator proposed by Sen and later extended by Hirsch was applied. This method calculates the median of pairwise rate-of-change estimates over time, allowing not only for the detection of a trend’s presence, but also for the determination of its direction and magnitude [87,88,89].

The linear trend is estimated using the median of all pairwise slopes:

(24) $$\;\beta =Median\left[\frac{x_{j}-x_{k}}{j-k}\right],\;k<j,$$

where 1 ≤ k < j ≤ n, and β is treated as the median of all possible pairs of combinations for the entire dataset.

This estimator provides a robust measure of the rate of change over time, making it suitable for datasets that may include non-normal distributions or outliers.

#### 3.2.4. Kolmogorov–Smirnov Test (KS)

The Kolmogorov–Smirnov (KS) test was used in this study as a statistical tool to assess the conformity of the correlation distributions—between entropy measures (ENTR, ∂ENTR/∂t, and |∇ENTR|) and extreme meteorological variables—to a normal distribution.

The primary goal of applying the KS test was to verify whether the spatial and seasonal distributions of the Spearman correlation values exhibited a shape consistent with the normal distribution, which is critical for properly interpreting the strength and nature of dependencies between the variables.

(25) $$D=\underset{x}{\sup}|F_{1}(x)-F_{2}(x)|$$

where $F_{1}\left(x\right),F_{2}(x)$ are the empirical cumulative distribution functions of the two samples; $\underset{x}{sup}$ denotes the maximum absolute difference between the two distribution functions.

## 4. Shannon Entropy as a Measure of Climate Information

Shannon entropy, though originally derived from information theory, is widely applied in environmental and climate data analyses as a nonparametric measure of uncertainty, disorder, and variability in probability distributions. In climatological literature, informational entropy has been successfully used to analyze seasonal and spatial climate variability, as well as to detect shifts in weather regimes. In the present approach, emphasis is placed on the empirical evaluation of entropy’s evolution across time and space as an indicator of local climate instability—without requiring reference to an external or idealized distribution [90]. Defining such a “reference” distribution can be challenging, if not impossible, in the context of complex and nonlinear atmospheric processes. Under conditions of high meteorological variability and spatial heterogeneity, adopting an arbitrary reference distribution may lead to ambiguous or misleading interpretations. It is important to note that Shannon entropy can serve as an indicator of the overall variability and instability of a distribution, but it should not be interpreted as a measure of the intensity or frequency of extreme events.

For this reason, a self-contained approach based on empirical entropy was adopted. This enabled tracking the degree of order in weather data without relying on additional model assumptions, thereby enhancing the method’s universality and robustness against errors arising from incorrect distributional specifications. In informational terms, an increase in Shannon entropy reflects a higher level of randomness and complexity in the distribution of climate variables, which directly translates into a reduced predictability of weather conditions and, potentially, greater vulnerability of the system to extreme events. Thus, the use of entropy as an indicator for analyzing climate variability aligns with the growing interest in nonparametric measures of uncertainty that allow for the assessment of irregularities and risk within a dynamically changing climate system.

Shannon entropy quantifies the uncertainty associated with predicting the value of a random variable [5,39,91]. It is calculated from the estimated probability distribution, and the accuracy of this estimate directly influences the reliability of the entropy computation. An improperly selected or poorly fitted distribution may result in erroneous entropy values, potentially leading to incorrect conclusions about the underlying climate structure.

The formula for the Shannon entropy of a continuous bivariate random variable (X,Y), with joint probability density function f(x,y), is defined as [38,92,93]:

(26) $$H_{S}\left(X,Y\right)=-\int_{R^{2}}f(x,y)\log_{2}f\left(x,y\right)dxdy$$

Here, $f\left(x,y\right)$ is the joint PDF of the bivariate distribution derived from the copula and marginals, marginal PDFs $f_{X}\left(x\right)$ and $f_{Y}\left(y\right)$. The copula density c(u,v) is derived from a selected family of copulas.

The formula for the joint PDF becomes:

(27) $$f\left(x,y\right)=c(F\left(x\right),G(y))f_{X}\left(x\right)f_{Y}\left(y\right)$$

Use the definition:

(28) $$H_{S}\left(X,Y\right)=-E(\log_{2}f\left(X,Y\right))$$

to estimate the entropy as the negative mean of the log joint PDF.

In discussing units of Shannon entropy for continuous distributions, the results are typically expressed in units of information—nats (when natural logarithms are used) or bits (when base-2 logarithms are used). The choice of unit depends on analytical conventions and the logarithmic system employed [12].

To preempt known criticisms and limitations associated with the use of Shannon entropy, this study was designed with methodological rigor. Measures implemented included:

- standardization of measurement units across the entire dataset,
- consistent estimation of marginal distribution parameters using the Maximum Likelihood Estimation (MLE) method,
- and uniform discretization procedures for all input data.

The selection of both marginal distributions and copula functions was guided by the Akaike Information Criterion (AIC), allowing for an objective assessment of model fit under consistent modeling assumptions. These methodological safeguards ensured the integrity of the entire procedure, aligning it with the rigorous standards required in climate data analysis—particularly in the context of studying extreme weather events and their relationship to informational measures of uncertainty, such as entropy.

One of the key limitations in using Shannon entropy as a measure of uncertainty in climate analyses is that the entropy of a probability distribution does not necessarily reflect the temporal predictability of a signal. It is possible for a meteorological variable (e.g., temperature or precipitation) to exhibit high entropy, suggesting substantial uncertainty about its values, while its temporal structure remains orderly and regular, making the signal highly predictable. Conversely, a signal with low entropy—which theoretically implies lower uncertainty—may, in practice, be chaotic if its values are irregularly distributed in time.

Such discrepancies arise because classical information entropy operates solely on frequency distributions, ignoring the temporal context and sequence dynamics. A particularly illustrative example is random time series shuffling (permutation): while such a permutation does not alter the entropy of the distribution (since the set of values and their probabilities remain unchanged), it completely destroys the temporal structure of the signal, resulting in a loss of predictability. This example highlights a critical limitation of the current approach based on static distributions.

Thus, while Shannon entropy is a powerful tool for analyzing spatial and structural variability, its temporal interpretation requires caution. There is a need to consider extending the approach to include dynamic or algorithmic entropy measures that account for sequence order and the properties of the underlying generating process.

The use of information entropy as a measure of uncertainty in climate variables, although theoretically well-founded and innovative, also involves important practical limitations, stemming from both the characteristics of the input data and the analytical methodology.

One major limitation is the spatial resolution of the data. A clear example is the comparative analysis between the NOAA and E-OBS datasets. Although grid harmonization (through spatial averaging or interpolation) was performed, such transformations inevitably introduce a degree of uncertainty—especially in regions with strong topographic variability (e.g., mountains and coastal areas), where local weather conditions can differ significantly over short distances.

A second important limitation concerns the temporal scale and data aggregation. While monthly aggregation facilitates comparisons and reduces random fluctuations, it can mask short-term extremes and dynamic weather changes—particularly relevant for convective precipitation, heatwaves, or frost events.

Moreover, entropy calculated from probability distributions estimated within a 70-year window for a given calendar month does not fully capture the intensity and frequency of extreme events occurring over shorter time intervals, which may limit its usefulness in the context of early warning for abrupt phenomena.

A third constraint involves the method of estimating the joint probability distributions, which directly affects the accuracy of Shannon entropy estimation. The use of long historical time series (e.g., 70-year moving windows) is justified from the perspective of statistical stability, but it can lead to the dilution of climate change signals, especially in the presence of non-stationarities or change points, as revealed by the Pettitt test. Consequently, entropy calculated over extended periods may not reflect the current structure of weather variability over shorter time horizons.

A fourth potential drawback is the method’s sensitivity to missing data and how these gaps are handled. Although NOAA data undergo homogenization and quality control, gaps may still occur in certain regions (e.g., over seas, sparsely populated, or mountainous areas), which are filled using various interpolation techniques. In regions with a high share of estimated data, entropy results may be subject to greater systematic error.

The combined effect of these limitations can reduce the accuracy of entropy estimation, particularly in localized analyses and at fine spatial scales. This may lead to the under- or overestimation of variability, which in turn can distort interpretations of trends and correlations with extreme weather events. On regional or continental scales, these limitations are less severe—results tend to retain greater statistical consistency and are useful for spatial comparisons. Nevertheless, the universality of findings should be treated with caution, especially when applied to local climate extreme forecasting or risk assessment in sensitive sectors, such as agriculture, water management, or disaster preparedness.

### 4.1. Entropy Fluxes as a Tool for Spatiotemporal Analysis of Climate Variability

In the analysis of extreme weather phenomena—such as heatwaves, droughts, or flash floods—information-theoretic metrics, particularly informational entropy, are increasingly being employed [61,94]. The proposed approach extends classical entropy analysis by incorporating field-based aspects: spatial gradients, divergence, and the associated entropy flux [95]. The introduction of operators known from continuum physics (such as ∇, ∇·, and ∇²) enables a mathematical representation of informational flows between adjacent cells of the analytical grid [96]. This serves as a foundation for detecting sources and sinks of climate variability. In particular, extreme events may be preceded by changes in the informational structure of the atmospheric system, whose spatiotemporal patterns can be captured through the analysis of entropy fluxes [17,97].

Informational entropy is not a classical energy function but rather a measure of informational disorder—making its diffusion conceptually different from classical heat diffusion. The interpretation of “information flux” as the spatial derivative of entropy is metaphorical but statistically valid, provided it is understood as a representation of statistical structure, rather than a literal physical energy transfer. It is essential to emphasize that in the context of “climate information”, the term refers to the statistical structure of weather variability, not to concrete datasets [24].

This study presents a framework based on the spatiotemporal analysis of informational entropy distributions aimed at understanding the dynamics of extreme climate events. Each geographic grid cell (with a resolution of 0.25° × 0.25°) is assumed to hold a value of entropy H(x,y,t), calculated from meteorological variables describing local conditions. This entropy can be interpreted as a measure of uncertainty or complexity of the climatic regime at a given location and time. The spatial gradient vector of entropy, ∇H(x,y,t), indicates the direction and intensity of uncertainty change across space, while its orientation reveals the direction in which structural changes in information propagate.

The entropy flux is then defined as:

$$J_{H}\left(x,y,t\right)=-D\cdot \nabla H\left(x,y,t\right)$$

representing a hypothetical flow of climate information between neighboring cells, analogous to diffusion mechanisms in physics. The divergence of this flux,

$${\nabla \cdot J}_{H}\left(x,y,t\right),$$

allows for the identification of areas that act as sources or sinks of variability, which can be critical in detecting spatial dynamic regimes. This formalism is grounded in classical field theory and employs well-established mathematical tools of vector calculus (gradient, divergence, Laplacian). Applying this methodology to meteorological data enables the identification of regions with elevated instability, which may serve as precursors to extreme weather events.

In particular, the temporal derivative of entropy, $\frac{\partial H}{\partial t}\left(x,y,t\right)$, serves as an indicator of local atmospheric system dynamics, where sharp increases may signal impending destabilization, such as intense precipitation or heatwaves. This approach effectively integrates classical concepts of information with climate process analysis and introduces a novel dimension to the detection and prediction of environmental hazards [60].

It should be emphasized that the interpretations of entropy gradients and fluxes presented here are hypothetical and serve as a conceptual framework for analyzing the dynamics of the climate system. Entropy values, their spatial gradients, and temporal derivatives are treated as quantitative indicators describing the system’s complexity and uncertainty; however, their links to atmospheric mechanisms—such as the persistence of local weather regimes or the generation of extremes—should be regarded as working assumptions that require further verification. In particular, the interpretation of the “stability of local weather regimes” refers to long-term statistical stability within the 70-year analysis windows, rather than a direct representation of short-term synoptic dynamics. We consider our approach as a starting point for further empirical and modeling studies that may confirm or refine the proposed relationships.

### 4.2. The Informational Entropy Field

For the informational entropy function derived from the bivariate distribution of temperature and precipitation variables (T,P) at a given spatial point (x,y), the time-window-based estimate can be expressed as:

(29) $$H\left(x,y,t\right)=H_{S}(T(x,y,t-∆t:t),P(x,y,t-∆t:t))$$

For each grid cell with geographical coordinates (x,y) at time t, the concept of the spatial entropy gradient vector is introduced as:

(30) $$\nabla H\left(x,y,t\right)=\left(\frac{\partial H}{\partial x},\frac{\partial H}{\partial y}\right)$$

where $\frac{\partial H}{\partial x},\frac{\partial H}{\partial y}$ denote partial spatial derivatives of entropy along the west–east and south–north axes, respectively. This gradient reflects both the direction and intensity of local informational complexity changes in space. The entropy flux vector is defined analogously to classical diffusion:

(31) $$J_{H}\left(x,y,t\right)=-D\cdot \nabla H\left(x,y,t\right)$$

where D is the entropy diffusion coefficient, which may be treated either as a fixed empirical constant or as a function of local environmental conditions.

Further analysis relies on the divergence operator; sources and sinks of entropy in space are identified via the divergence of the entropy flux vector:

(32) $${\nabla \cdot J}_{H}\left(x,y,t\right)=-D\cdot \nabla^{2}H\left(x,y,t\right)$$

when ${\nabla \cdot J}_{H}\left(x,y,t\right)>0$, positive divergence values indicate areas (grid cells) acting as sources of information, or generators of variability.

When ${\nabla \cdot J}_{H}\left(x,y,t\right)<0$, negative divergence values indicate absorbers of variability, associated with relative atmospheric stability.

### 4.3. Relationship with Weather Extremes

Climatic extremes can be analyzed through the lens of local entropy stability. Stability is quantified by examining temporal changes in entropy:

(33) $$\frac{\partial H}{\partial t}(x,y,t)$$

Persistently low entropy values $H\left(x,y,t\right)$ with weak spatial gradients suggest **a** stable weather regime—a potential predictor of droughts or heatwaves. Conversely, a sharp increase in the entropy time derivative $\frac{\partial H}{\partial t}$ signals regime destabilization, serving as a predictor of floods or severe storms.

The concept of entropy flux offers a novel perspective on climatic processes as a dynamic system of information exchange between neighboring regions. Areas with strong positive divergence may indicate localized instabilities conducive to extreme events—such as the initiation of convective storms or surface overheating under weak circulation conditions. In contrast, regions with negative divergence may correspond to stabilizing zones, for example, persistent high-pressure systems that sustain stable weather conditions.

This spatial differentiation enhances our understanding of which regions act as sources or sinks of variability over a given period, with direct implications for the risk assessment of extreme weather events.

Beyond spatial aspects, the temporal derivative of entropy $\frac{\partial H}{\partial t}$ provides insight into the stability of local weather regimes. An increase in this derivative can be interpreted as a signal of the destabilization and growing unpredictability of the system—conditions that often precede the onset of climatic extremes. The combined assessment of spatial entropy gradients and the temporal evolution of entropy and its derivatives delivers a more comprehensive picture of atmospheric system dynamics [98].

## 5. Results of the Analyses and Discussion

**Figure 1:**
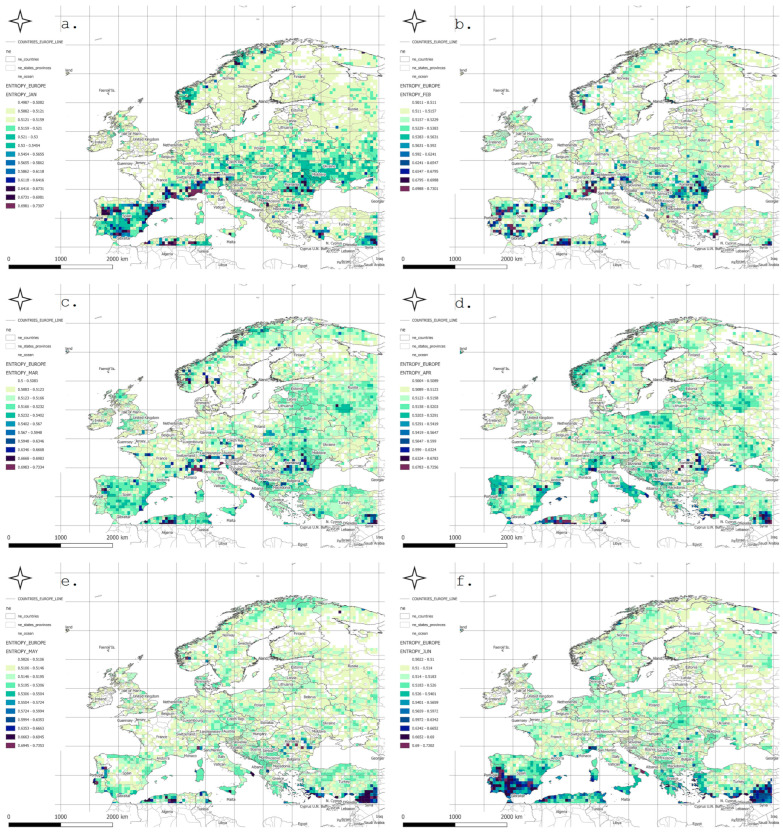

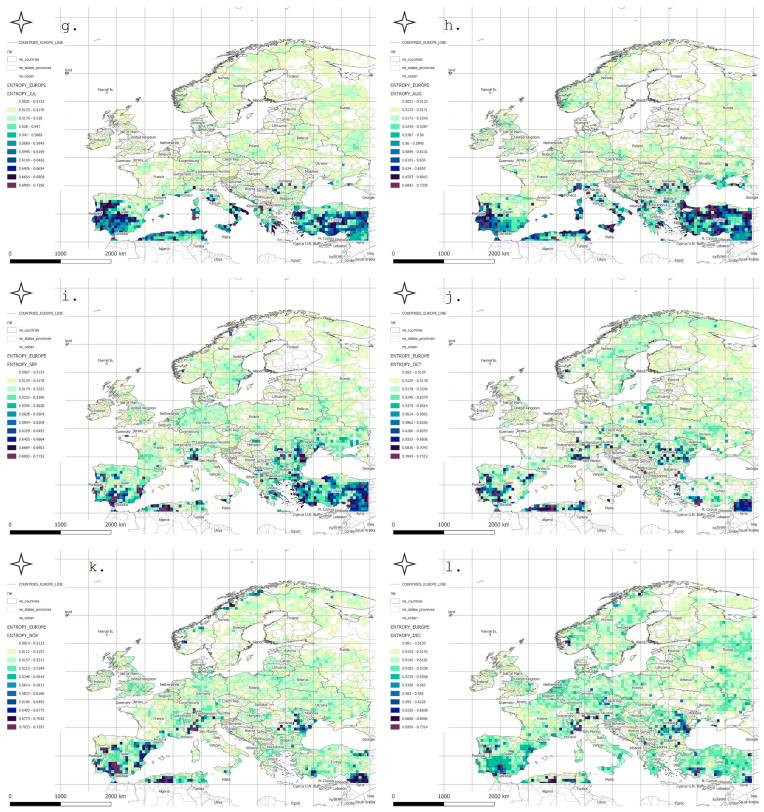
Seasonal Shannon entropy values [bits], calculated for the final period I–XII, 1941–2010. Each of the 12 panels (**a**–**l**) represents a calendar month, from January (**a**) (upper left corner) to December (**l**) (lower right corner). The color scale reflects entropy values: from the lowest (light yellow—low weather variability) to the highest (dark purple—high weather variability).

To enable a precise assessment of information entropy trends for precipitation and temperature, a block bootstrap resampling method was applied to generate multiple realizations of Shannon entropy. The entropy calculations were based on the bivariate joint probability distribution of temperature and precipitation (Figure 1).

The analysis of meteorological entropy was conducted separately for each calendar month, using data from the 1901–2010 climatological period. For each of the 40 years analyzed, entropy was calculated using 70-year seasonal time series: for the year 1971, the period 1901–1970 was used; for 1972, the period 1902–1971; and so on, up to 2010, for which data from 1941–2010 were used.

From the resulting time series of entropy values, seasonal trends were computed, and their statistical significance was evaluated using the Modified Mann–Kendall test (MMKT) at a 5% significance level. Additionally, to detect potential change points in the trend trajectories, the Pettitt test (PCPT) was applied, also at a 5% significance level. If the presence of a change point was confirmed, each newly segmented subsequence was analyzed separately for trends using the MMKT. If no change point was identified, the trend was assessed over the entire sequence. The results were presented graphically, providing a clearer and more precise visualization of the changes in entropy values and the evolution of their trends.

### 5.1. Shannon Entropy Values

For comparative purposes, the Shannon entropy values were normalized to the range [0, 1]. The original entropy range—computed via integration from the joint bivariate probability distribution based on a sample size of 70 (T, P) pairs—spanned from 0 to 12.259 bits. This normalization allowed for the consistent interpretation of spatial and temporal patterns of variability (Figure 1).

The maximum joint entropy for variables T and P was defined with respect to the number of possible states in a 70-element sample. For each marginal variable, the maximum entropy equals log(70), while for the bivariate distribution (T, P) it corresponds to (log(70)+log(70)=log(4900)). This value represents the case of a uniform distribution, in which all pairs (T, P) are equally probable, and serves as a reference point for normalizing the estimated Shannon entropy values. Through this normalization, entropy values fall within the range [0, 1], allowing for direct comparison across different grid points and time periods. In practice, values of Entropy(T, P) ≈ 1 indicate maximum uncertainty (a distribution close to uniform), whereas Entropy(T, P) ≈ 0 reflects a highly concentrated, ordered distribution.

In January, low entropy values dominated across Northern Europe, indicating stable and predictable winter conditions. Elevated entropy values appeared locally in the south—particularly over the Iberian Peninsula, southern France, and the Mediterranean regions—where precipitation variability is higher.

In February and March, similar patterns persisted, although the zone of increased entropy gradually shifted toward Central Europe. April showed growing spatial differentiation, particularly over the Alps, the Balkans, and Southeastern Europe. In May, a general decline in entropy was observed, reflecting the typical springtime stabilization of the atmosphere.

Entropy peaks in June and July, especially across Southeastern Europe—including the Balkans, Greece, Turkey, and Northern Africa—were likely linked to intensified convective storm activity and localized precipitation events. In August, high entropy zones shifted northward, covering parts of southern Germany, Poland, and Ukraine.

In September, variability was moderate in Western Europe, while remaining elevated in continental regions. October brought a further decline in entropy over Northern and Central Europe, though high values persisted in the Mediterranean basin. In November, entropy decreased across most regions, remaining moderate only in Southeastern Europe. December, like January, was marked by low entropy across nearly all of Europe, associated with dominant, stable winter synoptic patterns.

The observed spatial patterns of entropy revealed a pronounced seasonal rhythm: minimal variability during winter and peak variability during summer, especially in the southern and southeastern parts of the continent. High entropy in the warmer months reflected the intensification of convective processes and increased atmospheric instability.

Mountainous regions—such as the Alps and Carpathians—exhibited elevated entropy values throughout much of the year, indicative of complex orographic conditions and local microcirculations. In contrast, the Mediterranean coasts showed high variability particularly in autumn and winter, associated with episodic Mediterranean cyclones. Scandinavia and Northeastern Europe displayed the lowest entropy values for most of the year, confirming their stable and strongly seasonal climatic character.

### 5.2. Trend and Seasonal Variability of Shannon Entropy

**Figure 2:**
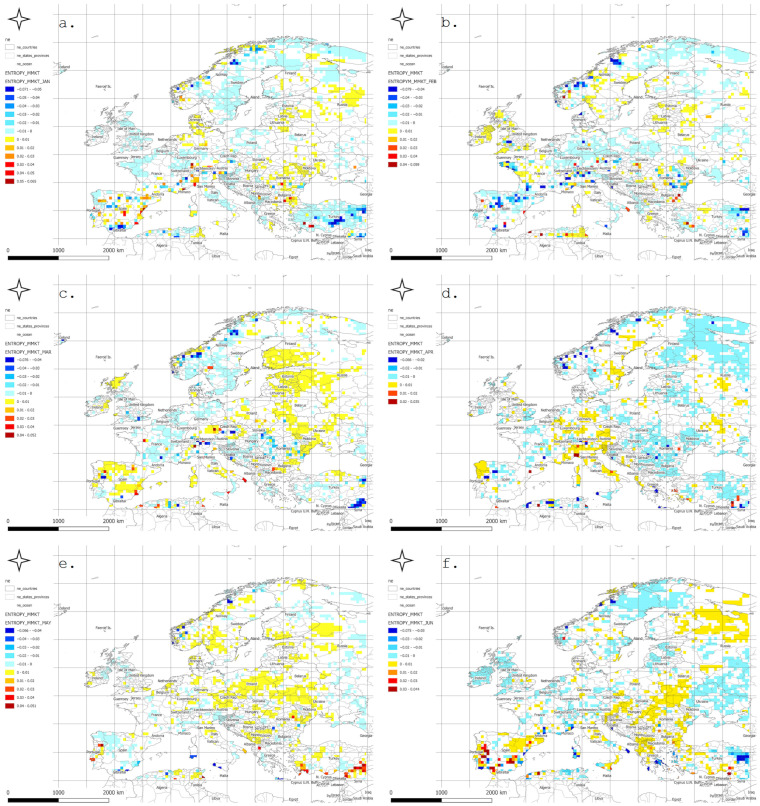

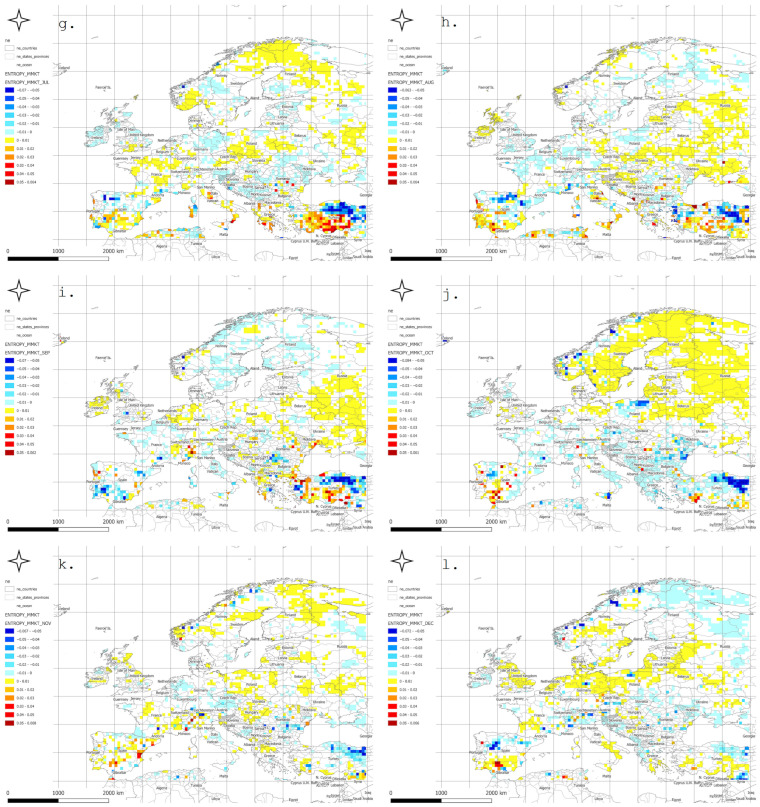
Results of the Modified Mann–Kendall Test (MMKT) at the 5% significance level, identifying seasonal trends in Shannon entropy values [bits/season], months I–XII. Each of the 12 panels (**a**–**l**) corresponds to a calendar month. The color scale illustrates the direction and magnitude of entropy trends: shades of red and orange indicate significant increasing trends, while shades of blue indicate decreasing trends. Statistical significance was assessed using the Modified Mann–Kendall Test at the 5% level.

The spatial distribution of meteorological entropy trends, calculated separately for each calendar month, is presented in Figure 2. In January, positive trends (yellows and light oranges) dominated, particularly across Central Europe and Scandinavia, which may indicate increasing variability in winter weather conditions. February displayed a greater number of negative trends in Southeastern Europe and the Alpine region, possibly reflecting a stabilization of local winter climates.

In March, distinctly positive trends appeared in Germany, Poland, Scandinavia, and northern France, suggesting a rise in weather variability during the early spring period. April presented a more spatially scattered trend pattern, though positive values still prevailed across Central and Eastern Europe.

May exhibited particularly strong positive trends in the Black Sea basin, the Balkans, and western Russia. June showed widespread positive trends across most of Europe, indicating a rise in atmospheric instability associated with the onset of summer.

In July and August, positive trends dominated Northern and Central Europe, while the southern parts of the continent showed more areas with no statistically significant changes. September revealed decreasing entropy trends in Spain, Portugal, and southern France, while positive trends persisted in the northern parts of the continent.

In October, entropy increases were especially prominent in Eastern Europe, including the Baltic states and western Russia. November brought marked positive trends in Central and Eastern Europe, along with localized decreasing trends in the western regions. In December, strong positive trends dominated across Northern Europe, with additional increases observed locally in the Alpine and Balkan regions.

**Figure 3:**
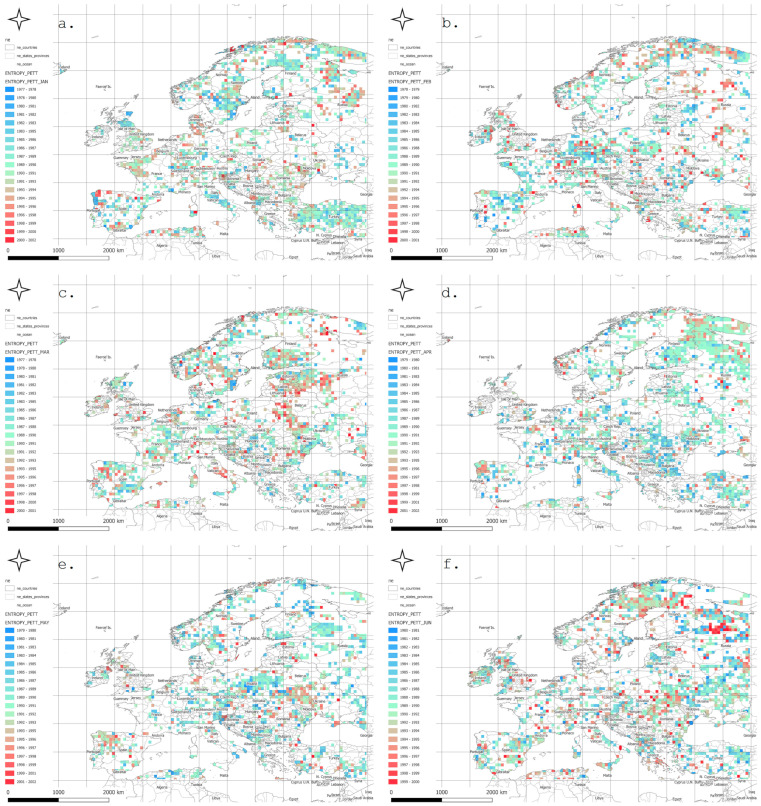

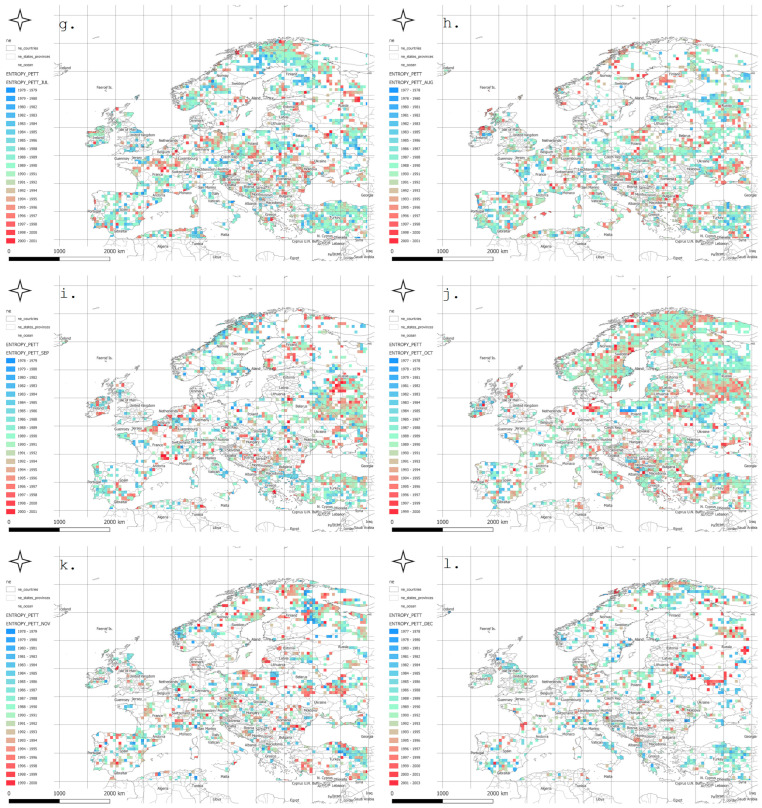
Results of the Pettitt change point test (PCPT) at the 5% significance level, identifying the years in which seasonal trends in Shannon entropy changed, months I–XII. Each of the 12 panels (**a**–**l**) corresponds to one calendar month—from January (upper left corner) to December (lower right corner). The colors on the maps indicate the year of the detected change point, ranging from 1971 (dark blue) to 2010 (red), while white areas denote grid points with no statistically significant trend changes.

Figure 3 presents the spatial distribution of the year in which a statistically significant change in meteorological entropy trend occurred, as determined using the Pettitt change point test (PCPT) at the 5% significance level.

In January, most change points were detected in windows ending after 1990, particularly across Central and Northern Europe. February showed a more dispersed pattern, with numerous change points in windows ending between 1980 and 1995—especially over the Balkans and southwestern Europe. In March, change points appeared earlier, often in windows ending in the 1970s, mainly in the Alpine region, Germany, and France. April exhibited a concentration of change points in windows ending around 1990, particularly in Central and Eastern Europe.

In May and June, red and orange hues dominated, indicating that the entropy trend changes tended to occur near the end of the analysis period (1995–2010). July and August displayed more spatially and temporally heterogeneous distributions of change points, suggesting that localized processes may be influencing entropy during the summer season.

In September, relatively early change points were observed, particularly in Northern Scandinavia and parts of Western Europe. October and November were characterized by a dense clustering of change points in Central and Northern Europe, primarily during the 1985–2000 period. December showed a prevalence of late-occurring changes, concentrated mostly in Southeastern Europe.

Mountain regions, such as the Alps and Carpathians, frequently exhibited earlier occurrences of change points, which may reflect a heightened sensitivity of entropy to shifting climatic conditions in orographically complex areas. In contrast, the absence of detected change points in regions such as Northern Scandinavia or Southern Spain suggests a relative stability of entropy trends in those areas.

The identified change point patterns also exhibited a seasonal character: during winter months, changes were more likely to occur in the final two decades of the study period, whereas during spring and autumn, the majority of change points clustered in the 1980s and 1990s.

### 5.3. Temporal Spearman Correlations

Below, we present correlation maps between meteorological entropy and selected climate parameters. The color scale on the right side of each map indicates the strength and direction of the correlation: warm colors (yellow, orange, red) represent positive correlations, while cool colors (various shades of blue) correspond to negative correlations.

#### 5.3.1. Spatial Relationship Between Entropy and Monthly Maximum Temperature

**Figure 4:**
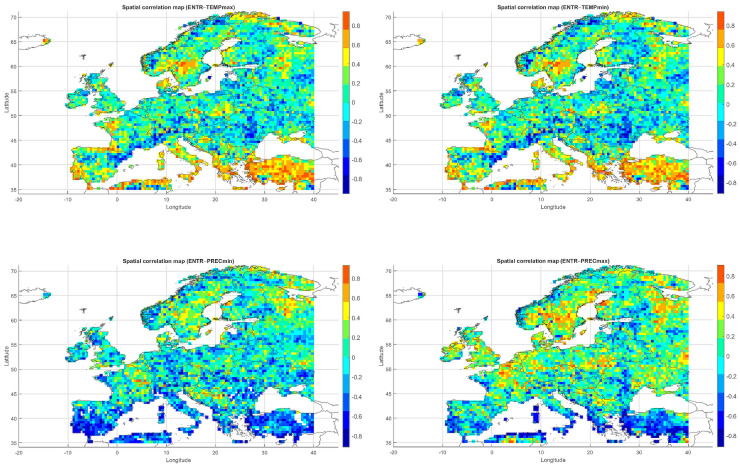
Spearman correlations between entropy and meteorological parameters.

The Spearman correlation map between informational entropy and monthly maximum temperature (Figure 4) revealed clear spatial variability in their relationship. In Southern Europe—particularly over the Iberian and Apennine Peninsulas, Greece, and Turkey—strong positive correlations dominated, locally exceeding 0.6. This suggests that increased atmospheric irregularity is associated with the occurrence of extremely high temperatures, which is typical of the Mediterranean climate regime.

In Central and Northern Europe (Germany, Poland, the Baltic States), correlations were weak or near zero, possibly reflecting the dominance of other climatic drivers. Scandinavia and the North Sea region also showed positive correlations, although more limited in extent. Mountain regions (the Alps, Carpathians, and Pyrenees) displayed strong local contrasts, likely influenced by orographic effects. A patchwork of correlations was observed in Eastern Europe (Ukraine, Russia), indicating the absence of a unified mechanism. In parts of France and Italy, notable negative correlations appeared, suggesting that higher entropy may coincide with lower maximum temperatures.

Overall, the spatial pattern indicates that the relationship between entropy and extreme temperatures is particularly pronounced in regions characterized by high seasonality and thermal instability.

#### 5.3.2. Spatial Relationship Between Entropy and Monthly Minimum Temperature

The correlation pattern with minimum temperature (Figure 4) differed significantly from that observed for $T_{max}$. In Southeastern Europe (Greece, Bulgaria, Turkey, and the Black Sea coast), strong negative correlations dominated, implying that higher entropy may be associated with nighttime cooling and an increased risk of low $T_{min}$ values.

In Central Europe, correlations were more variable—often close to zero, occasionally negative. In Scandinavia and northern Russia, weak positive correlations prevailed, which may reflect a buffering effect of atmospheric variability on temperature drops. Mountain regions (Alps, Carpathians) exhibited strong local contrasts, likely due to microscale processes and topographic complexity. Neutral relationships dominated in Western Europe (France, Germany), while the Iberian Peninsula displayed considerable heterogeneity, with both positive and negative values.

The relationship between entropy and $T_{min}$ is especially relevant in the context of frost risk and agricultural impacts. In arid and mountainous regions, strong negative correlations suggest the need for localized microclimatic analyses.

#### 5.3.3. Spatial Relationship Between Entropy and Monthly Maximum Precipitation

The map of entropy correlations with monthly maximum precipitation totals (Figure 4) revealed a predominance of positive correlations across Central and Northern Europe (Germany, Poland, the Baltic States, Scandinavia). This indicates that higher entropy may be linked to increased risk of high-precipitation months, often associated with cold fronts and cyclonic activity.

In Southern Europe (Spain, Italy, Greece), negative correlations prevailed, suggesting that in these regions, increased atmospheric variability does not correspond to greater monthly precipitation maxima—likely due to the influence of summer dry seasonality. Mountain areas (Alps, Pyrenees) displayed a diverse range of correlation values, reflecting local effects such as orographic lifting and convective storms. Western Europe (France, the British Isles) showed localized positive correlations.

In many temperate regions, correlations exceeded +0.4, indicating the potential of entropy as an indicator for extreme precipitation risk.

#### 5.3.4. Spatial Relationship Between Entropy and Monthly Minimum Precipitation

Correlations between entropy and monthly minimum precipitation totals (Figure 4) revealed a distinct north–south contrast. In Southern Europe (Spain, Italy, Greece, Turkey), strong negative correlations dominated, indicating a link between high entropy and increased drought risk. In Western and Central Europe, correlations were generally weak or neutral.

In Scandinavia and the British Isles, weak positive correlations prevailed, suggesting that atmospheric variability does not directly translate into dry months in these regions. Areas around the Black Sea exhibited negative correlations, likely due to strong precipitation seasonality and local circulation patterns.

In mountainous regions (Alps, Carpathians, Pyrenees), as well as in Southern Spain and Sicily, strong local negative correlations appeared, indicating that entropy may be a reliable indicator of monthly drought risk. Urban areas (e.g., London, Paris, Berlin) did not exhibit significant anomalies.

#### 5.3.5. Spatial Relationship Between Entropy Gradient Magnitude and Monthly Maximum Temperature

**Figure 5:**
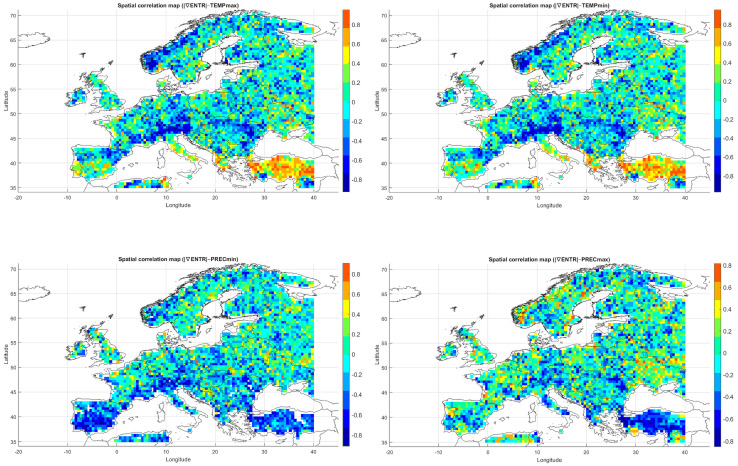
Spearman correlations between entropy gradient magnitude and meteorological parameters.

The Spearman correlation map between the entropy gradient and monthly maximum temperature (Figure 5) revealed strong regional contrasts. In Southeastern Europe (Turkey, Greece, and the Balkans), dominant strong positive correlations (>0.6) indicate a link between local variability changes and susceptibility to heatwaves. In these regions, the entropy gradient may reflect growing thermal instability.

In Central and Western Europe, correlations were predominantly negative or near zero, suggesting the stabilizing effect of a temperate climate. The Alps and southern Germany exhibited strong negative correlations, potentially indicating that increases in entropy gradients are associated with reductions in temperature extremes.

In Scandinavia (Norway and Sweden), positive correlations were observed, possibly reflecting the high sensitivity of the boreal climate. The Benelux countries, France, and the British Isles showed values close to zero, while localized positive correlations appeared in Italy, Ukraine, and Romania—regions that may serve as transitional zones between maritime and continental climatic influences.

Topography emerged as a significant factor: in mountainous areas, entropy gradients often displayed an inverse relationship with temperature compared to lowland regions.

#### 5.3.6. Spatial Relationship Between Entropy Gradient Magnitude and Monthly Minimum Temperature

The correlation map between the entropy gradient and minimum temperature (Figure 5) revealed strongly negative values in Southeastern Europe—particularly Turkey, Greece, and the Balkans—reaching below −0.6. This suggests that increasing variability may be associated with intensified cold nights, likely due to inversions or radiative cooling effects.

In Western Europe, correlations were near zero, while in the Alps and Carpathians, localized positive values appeared—likely influenced by terrain features and local atmospheric dynamics. Scandinavia exhibited moderate positive correlations, which may indicate the dampening of cold extremes under increased instability.

In the Adriatic region, the entropy gradient showed strong negative correlations with $T_{min}$, possibly driven by advective cooling. In Central Europe (Poland, Germany, Czechia), a mosaic pattern emerged, likely due to seasonal shifts in the influence of precipitation and snow cover.

The entropy gradient may serve as an indicator of frost risk, though the relationship is not uniform and requires seasonal analysis—particularly in winter months.

#### 5.3.7. Spatial Relationship Between Entropy Gradient Magnitude and Monthly Minimum Precipitation

The analysis of the entropy gradient’s correlation with minimum monthly precipitation (Figure 5) revealed a predominance of negative relationships in Southern Europe—especially in Spain, southern France, Greece, Italy, and Turkey. Values below −0.4 suggest a connection between increasing spatial variability and the risk of drought conditions.

In Central Europe (Poland, Czechia, Germany), correlations were weak or neutral. In Scandinavia and the North Sea region, positive correlations were more common, suggesting greater precipitation stability despite increased entropy gradients.

Localized positive correlations were observed in the Alps and Carpathians, while positive values dominated in Eastern Europe (Ukraine, Russia), indicating a more continental-type response of precipitation to spatial variability.

Overall, the entropy gradient appears useful in detecting regions vulnerable to drought, particularly in the Mediterranean basin.

#### 5.3.8. Spatial Relationship Between Entropy Gradient Magnitude and Monthly Maximum Precipitation

Correlations between the entropy gradient and monthly maximum precipitation (Figure 5) were generally positive across temperate regions—especially in Germany, Poland, the Baltic States, and Western Russia—ranging from 0.2 to 0.6. This suggests that spatial instability is associated with the intensification of extreme precipitation episodes.

Scandinavia (Norway and Sweden) also showed positive correlations. In Southern Europe (Spain, Italy, Greece), correlations were mostly negative or neutral, likely due to strong seasonality and limited spatial variability during dry periods.

Urban areas (e.g., London, Paris, Berlin) and mountain regions (e.g., the Alps) exhibited moderate positive relationships. The entropy gradient thus emerges as a sensitive indicator of spatial rainfall dispersion, potentially valuable for assessing flash flood risk.

### 5.4. Seasonal–Spatial Spearman Correlations

A spatial–seasonal analysis was performed on the maximum absolute values of the Spearman rank correlations between meteorological entropy (incorporating monthly temperature and precipitation) and extreme climate parameters. Time lags from 1 to 12 months were considered to evaluate the predictive potential of entropy in anticipating climate changes.

#### 5.4.1. Entropy and Monthly Minimum Temperature 

**Figure 6:**
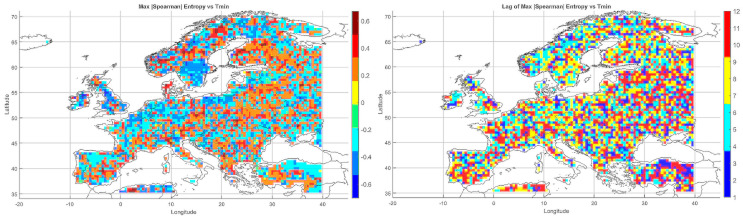
Maximum absolute Spearman correlation $max\left|\rho (Entropy,T_{min})\right|$, statistically significant at the 5% level.

Figure 6 presents maps of the maximum absolute correlations (left panel) and the corresponding time lags (right panel). In Central-Eastern Europe and Scandinavia, positive correlations dominated (ranging from 0.4 to 0.6), suggesting that increases in entropy precede declines in $T_{min}$. In contrast, negative correlations were observed in Southern Europe (Spain, Italy, Greece), indicating the inverse relationship.

The most frequent lag values fell between 6 and 10 months, pointing to the seasonal nature of the observed interactions. In higher latitudes (above 60° N), correlations remained positive, with shorter lags (3–5 months) reflecting the more immediate response of boreal climates. The spatial consistency of 6–9 month lags across Central-Eastern Europe may indicate the presence of a semiannual climatic memory mechanism.

#### 5.4.2. Entropy and Monthly Maximum Temperature

**Figure 7:**
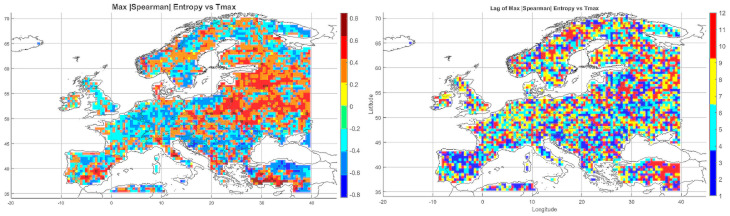
Maximum absolute Spearman correlation $max\left|\rho (Entropy,T_{max})\right|$, statistically significant at the 5% level.

Figure 7 presents an analogous analysis for $T_{max}$. Strong positive correlations (>0.4) were observed in Central-Eastern Europe and Scandinavia, suggesting that entropy is a reliable indicator for forecasting episodes of elevated temperatures. In Southern Europe, negative correlations dominated, possibly reflecting local damping mechanisms.

The strongest associations occurred with time lags of 6–9 months. Western Europe showed greater spatial variability and generally weaker correlations, likely due to oceanic influence and seasonal instability. The similarity in lags between $T_{min}$ and $T_{max}$ points to the seasonal consistency of the system’s response. However, regional differences in correlation strength and direction highlight the need for localized modeling approaches for maximum temperatures.

#### 5.4.3. Entropy and Monthly Minimum Precipitation

**Figure 8:**
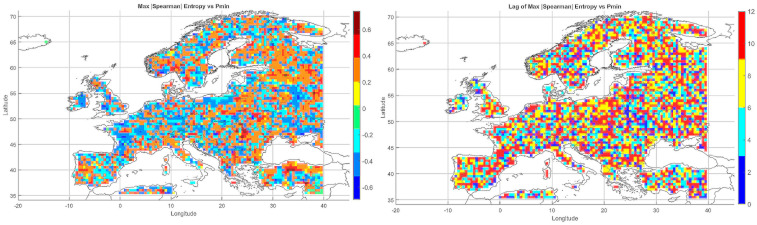
Maximum absolute Spearman correlation $max\left|\rho (Entropy,P_{min})\right|$, statistically significant at the 5% level.

Figure 8 illustrates the spatial distribution of the correlations and time lags between entropy and $P_{min}$. Positive correlations (>0.4) prevailed across Central and Northeastern Europe, indicating that entropy anomalies precede periods of reduced rainfall. In Southern Europe (Spain, Greece, Turkey), negative correlations (down to −0.6) dominated, suggesting distinct climatic dynamics in these regions.

The strongest correlations occurred at lags of 6–10 months, while in Southern Europe, the lags were shorter (<4 months). Eastern Europe exhibited a high degree of lag regularity, potentially indicating a stable climatic rhythm. In countries such as Poland, Ukraine, and Finland, entropy may serve as a strong predictor of drought conditions. Variability in mountainous areas may be influenced by orographic effects.

#### 5.4.4. Entropy and Monthly Maximum Precipitation

**Figure 9:**
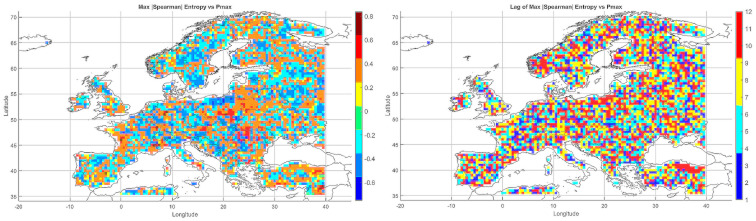
Maximum absolute Spearman correlation $max\left|\rho (Entropy,P_{max})\right|$, statistically significant at the 5% level.

Figure 9 presents the relationship between entropy and monthly precipitation maxima. Positive correlations (>0.4) dominated across Central and Eastern Europe, with local maxima up to 0.8 observed in southern Germany, Austria, and the Czech Republic—where increased entropy preceded intense precipitation events.

The strongest correlations were found at the 6–9 month lags, with several regions also showing peaks at 12 months, suggesting the existence of annual precipitation cycles. In the Mediterranean basin, correlations were weaker or even negative, likely due to seasonal dryness and irregular atmospheric dynamics. High correlations in mountainous regions (Alps, Carpathians) suggest that topography significantly modulates the precipitation response to entropy variability.

Meteorological entropy showed strong delayed associations with extreme values of temperature and precipitation. The highest positive correlations were observed in Central Europe, Scandinavia, and parts of Eastern Europe. The most common time lags were in the 6–9 month range, indicating a seasonal translation of entropy variability into climate parameters. Topography and local factors (e.g., oceanic climate, seasonal circulation) significantly influence the strength and direction of these relationships. Entropy may function as an early proxy for predicting $T_{max},\;T_{min},\;P_{max},\;P_{min}$, offering potential applications in climate modeling and early warning systems for extreme weather events.

#### 5.4.5. Temporal Derivative of Entropy and Minimum Temperature

**Figure 10:**
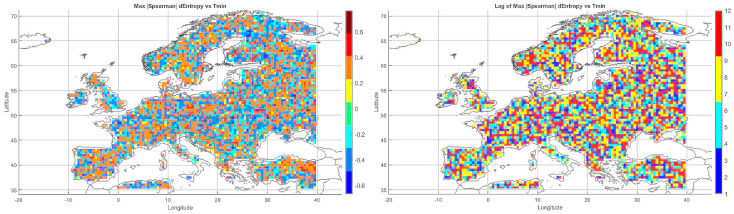
Maximum absolute Spearman correlation $max\left|\rho (dEntropy,T_{min})\right|$, statistically significant at the 5% level.

Figure 10 displays the maximum absolute Spearman correlations between the temporal derivative of entropy and monthly minimum temperature, along with the corresponding time lag. The left panel shows the correlation strength, and the right panel shows the lag (in months) at which the correlation peaks.

High positive correlations (up to 0.6) were observed in Central and Eastern Europe (Poland, Germany, Ukraine, western Russia), suggesting a strong seasonal link between entropy change and $T_{min}$ Scandinavia also exhibited significant positive correlations, confirming the responsiveness of colder climates to systemic variability.

In mountainous regions (Alps, Carpathians), correlations were more variable, likely due to orographic influences. In Southern Europe (Greece, southern Italy), negative correlations were present, probably associated with distinct thermal regimes. Most common lags ranged from 5 to 9 months, indicating a seasonal nature in $T_{min}$ response to entropy changes. In some areas, a lag of 12 months was observed, suggesting annual cycles in climatic response. Shorter lags (2–4 months) in Southern Europe may indicate quicker responses to atmospheric anomalies.

#### 5.4.6. Temporal Derivative of Entropy and Maximum Temperature

**Figure 11:**
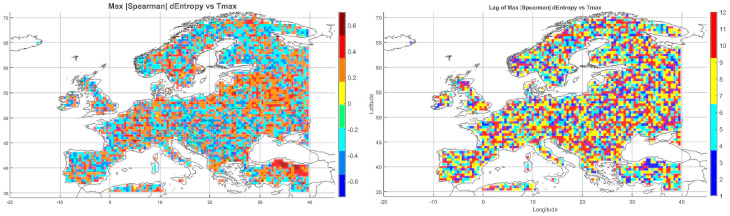
Maximum absolute Spearman correlation $max\left|\rho (dEntropy,T_{max})\right|$, statistically significant at the 5% level.

Figure 11 shows the correlation between the temporal derivative of entropy (dEntropy) and monthly maximum temperature ($T_{max}$), with time lags included.

Positive correlations (0.2–0.6) dominated in Central and Eastern Europe (Poland, Czechia, Germany, western Russia), indicating a relationship between rapid changes in atmospheric variability and extreme temperatures. In Greece, Turkey, and the Balkans, correlations reached up to 0.65, likely due to heatwaves and Mediterranean convective circulation.

In Western Europe (France, the UK), correlations were weaker and more scattered—likely influenced by oceanic climate. Mountainous and coastal areas showed greater correlation variability, reflecting local topographic and hydrological conditions.

Most common lags ranged from 6 to 9 months, particularly in Central Europe. Longer lags (10–12 months) in the Baltic States and Finland may indicate a cumulative thermal response. Shorter lags occurred mainly in coastal regions.

#### 5.4.7. Temporal Derivative of Entropy and Monthly Minimum Precipitation

**Figure 12:**
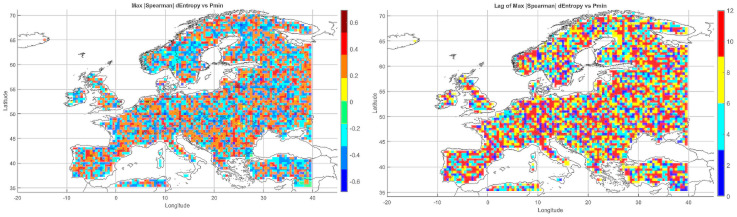
Maximum absolute Spearman correlation $max\left|\rho (dEntropy,P_{min})\right|$, statistically significant at the 5% level.

Figure 12 presents the spatial distribution of maximum correlations between dEntropy and monthly minimum precipitation sums.

Positive correlations (0.4–0.6) dominated in Central and Eastern Europe (Poland, Germany, Ukraine, Belarus), suggesting that rising climate system variability may precede periods of rainfall deficit. Western Europe and Scandinavia showed weaker correlations, possibly due to higher weather variability and oceanic influence.

Southern Europe displayed more spatially heterogeneous correlations. Most common lag values ranged from 6 to 10 months, though in parts of southern and western Europe, lags were shorter (2–5 months). This suggests regional differences in climate sensitivity to entropy changes.

Lag analysis indicates that dEntropy could serve as a useful early warning indicator for seasonal drought conditions.

#### 5.4.8. Temporal Derivative of Entropy and Monthly Maximum Precipitation

**Figure 13:**
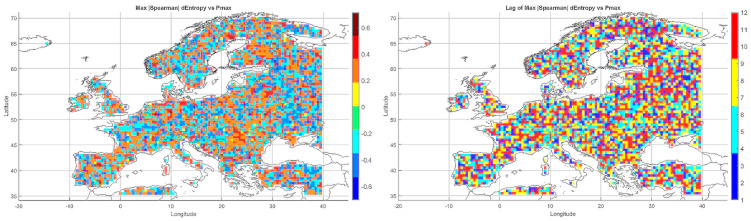
Maximum absolute Spearman correlation $max\left|\rho (dEntropy,P_{max})\right|$, statistically significant at the 5% level.

Figure 13 illustrates the correlations between the temporal derivative of entropy and maximum monthly precipitation totals.

The strongest positive correlations (up to 0.6) occurred in Central-Eastern Europe (Poland, Czechia, Ukraine, Romania), indicating that dEntropy may be an effective predictor of intense rainfall episodes. Western Europe and Scandinavia showed significantly weaker correlations.

Southern Europe (Spain, Greece, Italy) exhibited mixed relationships—both positive and negative—highlighting greater regional complexity. High correlations in the Carpathians, Alps, and Balkans likely reflect local orographic effects promoting convection.

Most frequent lag values fell between 6 and 10 months. In Southern Europe, shorter lags (2–5 months) dominated, possibly due to faster atmospheric responses. The high spatial coherence of lag patterns suggests the presence of shared circulation mechanisms.

### 5.5. Entropy Relationships with Climate Indices ENSO Oraz GLBSST

Informational entropy, calculated from meteorological variables (temperature and precipitation), is increasingly applied as an indicator for analyzing the variability and non-stationarity of climate systems—both at regional and global scales. Particularly noteworthy is its potential to reflect the influence of processes such as global warming (e.g., GISTEMP index) and oceanic oscillations (e.g., ENSO—El Niño Southern Oscillation).

Due to its sensitivity to changes in atmospheric dynamical structure, entropy may complement classical circulation indices by providing additional insight into the underlying mechanisms of weather systems. The presence of strong positive correlations across Eastern and Northern Europe suggests that entropy may serve as an indirect proxy for sea surface temperature (SST) anomalies, while negative correlations in regions such as the Mediterranean may indicate local hydrological anomalies or differing modes of climate response.

#### 5.5.1. Entropy and GLBSST (GISTEMP)

**Figure 14:**
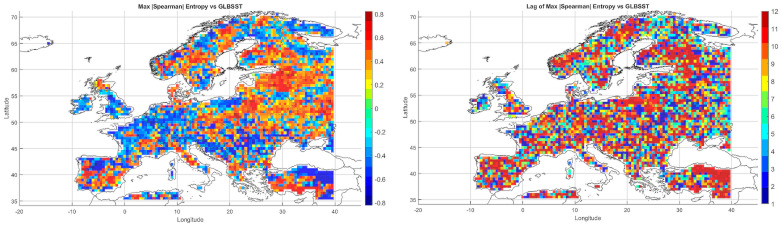
Maximum absolute Spearman correlation $max\left|\rho (Entropy,GLBSST)\right|$, statistically significant at the 5% level.

Figure 14 presents the maximum absolute values of the Spearman correlations between informational entropy derived from meteorological fields and the global sea surface temperature index GLBSST (GISTEMP), along with the corresponding time lags.

The left panel shows that positive correlations dominated across Central, Eastern, and Northern Europe, reaching values above 0.6 in some areas. This suggests a strong influence of global SST fluctuations on continental climate variability.

In Southern Europe—particularly in Turkey and northern Africa—correlations were predominantly negative, which may reflect a distinct mechanism for GLBSST signal transmission within the Mediterranean climate regime.

The right panel displays the spatial distribution of time lags, which most frequently ranged from 9 to 12 months. This is consistent with established mechanisms of energy transfer between ocean and atmosphere. The homogeneity of lag times across Central and Eastern Europe contrasts with greater variability in the south, highlighting the potential role of local topographic conditions in modulating atmospheric response.

#### 5.5.2. Entropy and ENSO (NINO3.4)

**Figure 15:**
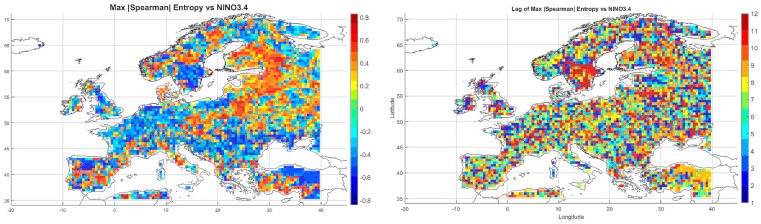
Maximum absolute Spearman correlation $max\left|\rho (Entropy,NINO3.4)\right|$, statistically significant at the 5% level.

Figure 15 illustrates the relationships between meteorological entropy and the ENSO index NINO3.4, including the time lags at which the correlations are strongest. High positive correlations (exceeding 0.6) were observed across Scandinavia, Russia, and Central Europe, confirming the influence of ENSO on climate variability in these regions despite their considerable distance from the tropics.

In Southern Europe—including the Mediterranean basin, Turkey, and the Iberian Peninsula—negative correlations dominated, suggesting an inverse ENSO effect in the warm, arid climates of the southern continent.

The distribution of time lags shows that the strongest correlations typically occurred with lags of 8–12 months in Eastern and Northern Europe, indicating a long-term, indirect ENSO influence via atmospheric circulation mechanisms. In contrast, Western and Southern Europe exhibited shorter lags (4–6 months), suggesting a faster atmospheric response.

Meteorological entropy showed statistically significant relationships with global climate indices, both in terms of strength and spatiotemporal structure. Both GISTEMP and ENSO influenced weather variability in Europe with notable time lags, making entropy a potentially valuable prognostic indicator—especially in early warning systems for climate anomalies. The observed regional differences in correlation magnitude and lag length underscore the need for local calibration and the integration of entropy into seasonal forecasting models alongside established climate indices.

### 5.6. Entropy Fluxes—Localization of Entropy Sources and Sinks

Informational entropy fluxes and their associated sources and sinks are closely linked to prevailing atmospheric circulation types over Europe.

Western circulation patterns (types W, SW) favor west-to-east entropy transport, reflecting the activity of Atlantic low-pressure systems. Anticyclonic blocking events (e.g., NE patterns, continental regimes) are associated with the formation of "entropy holes"—areas of reduced weather variability, particularly over Central and Eastern Europe. Southern circulation (S, SE) is linked to local entropy sources over the Mediterranean and Balkans, driven by convection and the influx of moist, unstable air masses.

Elevated spatial entropy often accompanies the passage of atmospheric fronts, especially under strong thermal gradients.

Regions exhibiting locally elevated entropy can be interpreted as generators of climatic variability, often located in dynamic transitional zones such as:

- The Iberian Peninsula,
- The Balkans,
- Scandinavia.

In contrast, entropy sinks—areas where entropy is lower than the surrounding environment—tend to exhibit greater atmospheric stability and are more susceptible to external disturbances. Analyzing these structures facilitates the identification of initiation points and propagation pathways of extreme weather events.

**Figure 16:**
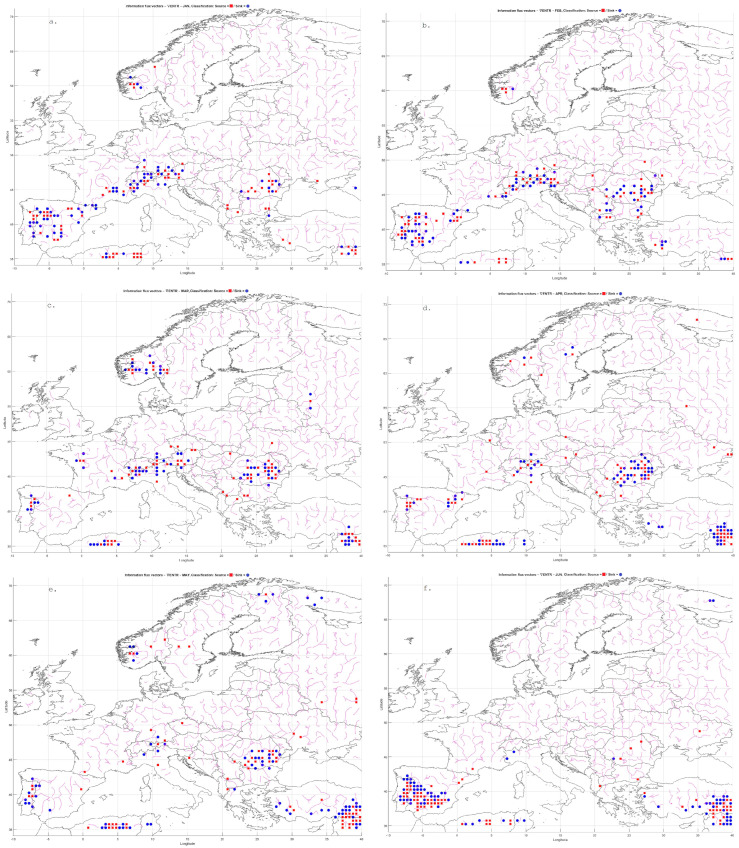

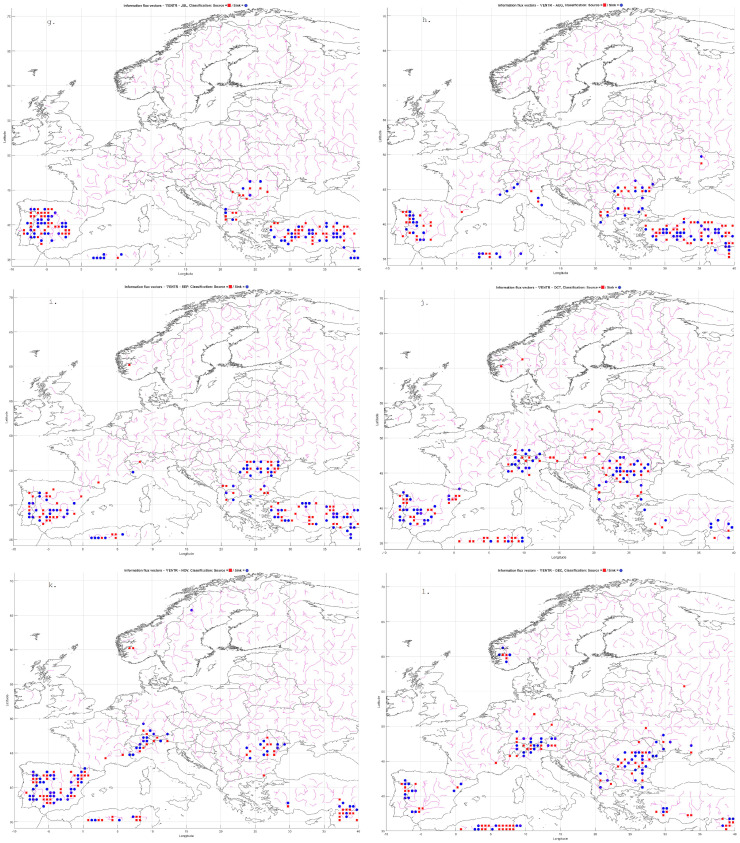
Shannon entropy fluxes (pink) for temperature and precipitation over Europe, months I–XII, year 2010. Each of the 12 panels (**a**–**l**) corresponds to one calendar month—from January (upper left corner) to December (lower right corner). The figure highlights the sources (red squares) and sinks (blue circles) regions of information entropy.

Figure 16 presents, for each calendar month, the vector fields of information entropy flux, calculated from the spatial gradient of the normalized entropy field. The streamlines illustrate both the direction and the relative intensity of climate information flow at the mesoscale.

Seasonal flow patterns include:

- Winter (January–February): Dominated by organized flows from the north and northeast, especially over Scandinavia and Eastern Europe, associated with continental air masses and stable pressure systems.
- Spring (March–April): Increasing local variability and more diversified flow structures reflect the transitional nature of the season.
- Summer (July–August): Characterized by short, meandering entropy streams, indicating enhanced local turbulence. In Southern Europe, more organized westward flows appear, linked to land–ocean thermal gradients.
- Autumn (September–November): Flows begin to reorganize toward wintertime patterns. May shows intensification over the Balkans, while September highlights increased activity over Western Europe.

Strong entropy sources and sinks—identified as divergences and convergences of entropy streamlines—frequently appear seasonally over Central Europe, suggesting its role as a nodal region for the transmission of climate signals. These structures are essential for understanding the regional spread of weather disturbances. To identify thresholds for delineating source and sink regions, the 5th and 95th percentiles of $\nabla \cdot J_{H}$, were determined and are marked in Figure 16 separately for each month.

Spatial patterns of entropy fluxes demonstrated correlations with the global teleconnection indices:

- Both NAO and ENSO appear to modulate the direction and intensity of atmospheric information transport.
- Months such as March and November exhibited highly directional flows, indicating the dominance of specific circulation mechanisms.
- In other months, more chaotic flows prevailed—typical of local diffusion and convective dynamics.

Topography plays a crucial role in shaping these structures. The Alps, Carpathians, and Scandinavian Mountains often act as barriers to the propagation of climate information, influencing the configuration and behavior of entropy fluxes across the continent.

**Figure 17:**
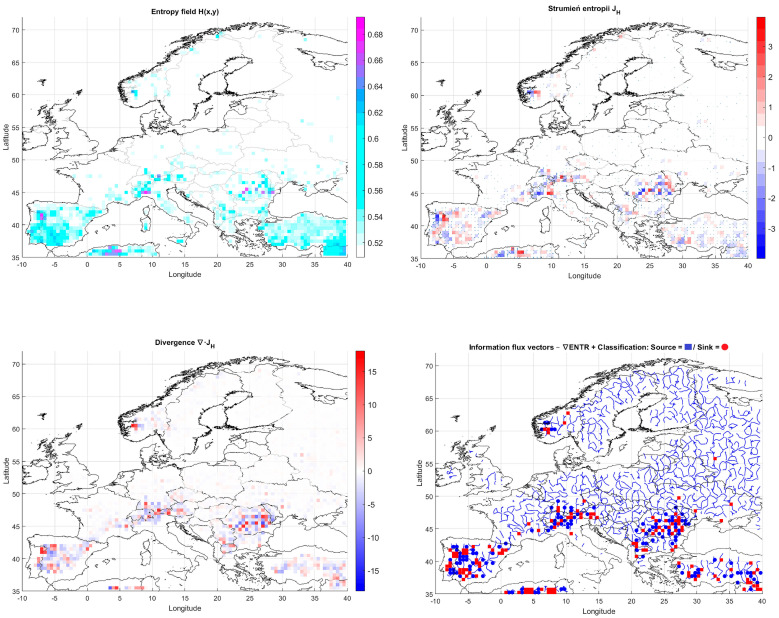
Spatial–temporal components of informational entropy in the climate-scale analysis over Europe for 2010. The figure highlights the sources (red squares) and sinks (blue circles) regions of information entropy.

The maps in Figure 17 illustrate the key components of the spatial–temporal analysis of informational entropy at the climatic scale across Europe. The top-left panel presents the entropy field H(x,y), representing the local complexity of weather variability as measured by Shannon entropy, calculated from the joint distributions of temperature and precipitation. 

The highest entropy values, indicating the greatest uncertainty in weather regimes, were observed in the Mediterranean basin and the southern Balkans, suggesting significant seasonal instability in these regions. In contrast, Northern and Central Europe exhibited low H(x,y), values, which implies a greater predictability of climatic patterns, potentially linked to the dominance of stable barometric systems.

The top-right panel shows the entropy flux $J_{H}$ (a vector field of information transfer), computed as the negative gradient of the H(x,y) field. Areas with strong $J_{H}$ values, particularly over the Pyrenees, Alps, and Carpathians, indicate substantial climatic information transfer between adjacent grid cells—likely resulting from orographic influences and localized atmospheric circulation.

The bottom-left panel depicts the divergence $\nabla \cdot J_{H}$, identifying source regions (positive values) and sink regions (negative values) of entropy. Notably high positive divergence appeared over southern France, Switzerland, and parts of Italy, suggesting these as localized centers of weather variability generation. Conversely, negative divergence values over Hungary and Romania designated these areas as entropy sinks—absorbing external climatic signals.

To delineate neutral, source, and sink regions, threshold values were defined based on the 5th and 95th percentiles of $\nabla \cdot J_{H}$: the lower threshold (sink) at −3.464, and the upper threshold (source) at 3.646. The classification shown in the bottom-right panel assigned each grid cell to one of three categories: source (1), neutral (0), or sink (−1). A clear dominance of the neutral state was observed, consistent with expectations for most regions experiencing relatively homogeneous and stable weather conditions. The notable concentration of sources in mountainous regions emphasizes the role of topography in generating local entropy.

The spatial analysis of the informational entropy field revealed structured variability across Europe at the continental scale. High entropy values clustered over southern and eastern Europe, highlighting areas of elevated disorder and potential atmospheric instability. The entropy field displayed a clear directional structure, indicating paths of fluctuation propagation. Entropy fluxes revealed the dominant spatial pathways of weather information transport, strongly aligned with prevailing circulation trajectories. A predominant west-to-east and south-to-central continental flux was observed, reflecting the combined influence of oceanic air masses and convective activity in southern regions. Local clustering of streamlines points to so-called “information nodes”—regions with high potential for information exchange.

The divergence field captures zones of information accumulation and dissipation, offering crucial insight into identifying climatic instability sources and sinks. Areas with positive divergence are interpreted as sources—regions initiating climate variability—while negative divergence denotes zones that suppress instability (entropy sinks). A clear seasonal signature of sources and sinks is also evident, which may be of prognostic significance. Source regions often precede the emergence of extremes, whereas sink zones may act as buffers against atmospheric disturbances. Spatial classification also facilitates the identification of transitional zones where local dynamics can undergo rapid shifts.

The persistent occurrence of entropy sources in southeastern Europe may indicate a heightened climatic sensitivity to external drivers such as ENSO. The spatial coherence of entropy fluxes and divergence fields supports the validity of the applied mathematical formalism. This methodological framework enables not only the visualization of the static field of weather complexity, but also the quantitative capture of the directions and centers of atmospheric information transmission.

### 5.7. Coincidence of Extreme Weather Events with Selected Features of Informational Entropy Transport

Extreme weather phenomena—such as heatwaves, droughts, and convective storms—demonstrate significant relationships with the spatial and temporal characteristics of informational entropy transport. Several key features play a critical role in this dynamic:

- **Entropy sinks**—regions of negative divergence in the entropy flux field ($\nabla \cdot J_{H}\;<\;0$), where information streamlines converge. These areas are frequently associated with persistent anticyclonic blocks, atmospheric stagnation, and the occurrence of droughts or heatwaves. In such zones, the climate system exhibits a reduced capacity for further dynamical evolution.
- **Entropy sources**—areas of positive divergence ($\nabla \cdot J_{H}>\;0$), where information fluxes diverge radially outward. These are often dynamic convective zones conducive to the generation of intense thunderstorms, convective precipitation, and hailstorms. Notable source regions include the Balkans, the Alps, the Caucasus, and the eastern Mediterranean.
- **Entropy fronts**—regions marked by strong spatial gradients (|∇H|) and discontinuities in the entropy field. These structures typically emerge along frontal zones, at the boundaries between air masses, and over mountainous terrain. They frequently act as initiators of severe weather events, such as downpours and convective storms.

Seasonal Analysis Findings:

- In summer, localized entropy sources (e.g., over the Balkans or Mediterranean coasts) often precede the onset of storms and intense rainfall events.
- In winter, entropy sinks across Central Europe and Scandinavia correlate with cold spells and dry periods.
- In mountainous regions (such as the Alps or Carpathians), the presence of so-called “entropy holes” may favor the development of closed convective storm systems.
- Areas characterized by high entropy gradients (e.g., Scandinavia, Russia) often temporally precede episodes of winter weather extremes.

Western and southern circulation regimes (W, SW, S) tend to favor the emergence of entropy sources and intensify information fluxes. In contrast, continental high-pressure systems (NE types, anticyclones) contribute to the closure of entropy streamlines, leading to the formation of sinks and an elevated risk of weather stagnation.

Sudden increases in temporal entropy values (∂H/∂t) may indicate seasonal transitions—such as the shift to a wet summer or dry autumn. These inflection points often precede the occurrence of extreme events and are consistent with trend detection methodologies (Mann–Kendall and Pettitt tests).

The occurrence of entropy sources, sinks, and fronts is linked to broader systemic signals, including:

- **ENSO (El Niño–Southern Oscillation)**—particularly during El Niño phases, an increase in entropy source activity is observed over southern Europe, accompanied by intensified extreme precipitation.
- **GISTEMP (Global Sea Surface Temperature Index)**—correlates with entropy anomalies, especially in the Mediterranean and Eastern European regions.

### 5.8. Prognostic Significance

This analysis confirms that the spatial structure of informational entropy can serve as an early indicator of forthcoming weather extremes. Entropy values and their derivatives (∇H, ∂H/∂t) tend to lead the temporal peaks of extreme temperatures ($T_{max},\;T_{min}$) and precipitation ($P_{max},\;P_{min}$).

Locations exhibiting strong gradients, convergence zones, and divergence structures in entropy fluxes form a predictive framework that can be applied in:

- seasonal forecasting models,
- early warning systems,
- regional climate risk assessments.

The characteristics of entropy transport—such as sinks, sources, discontinuities, and temporal spikes—are closely tied to the manifestation of extreme weather events across Europe. Entropy streamlines reflect the dynamical organization of atmospheric systems and may serve as functional prognostic indicators. The integration of informational entropy into climatology opens new avenues for diagnosing weather instability and for adaptive climate risk management strategies.

### 5.9. Comparative Analysis of Correlations

Table 3 presents a comparative overview of the spatially distributed, maximum temporal Spearman correlations between measures of entropy (ENTR) and its spatial gradient (|∇ENTR|) with four key meteorological variables: maximum temperature, minimum temperature, maximum precipitation, and minimum precipitation. The Kolmogorov–Smirnov test for distribution conformity unambiguously indicates that the correlation distributions deviated significantly from normality (*p* < 10^−11^). This suggests that the information transfer between entropy and climatic extremes is inherently nonlinear and exhibits strong spatial heterogeneity.

In addition, Shapiro–Wilk tests were conducted, and in all analyzed cases the results were negative, indicating a lack of conformity between the examined distributions and the normal distribution. The findings clearly show that the spatial and seasonal distributions of correlation values are non-normal. This implies that classical methods based on the assumption of normality (e.g., parametric tests relying on means and variances) are not appropriate for analyzing these data.

Instead, it is necessary to apply nonparametric methods that are robust to deviations from normality, such as Spearman’s rank correlation, nonparametric significance tests, or bootstrap procedures. This characteristic of the data suggests that the observed relationships are shaped by nonlinear processes and strong spatial heterogeneity, further supporting the use of entropy- and information-based measures.

A comparison of the distributions of ENTR and its gradient revealed statistically significant discrepancies, confirming that the flow of information between the two is complex and non-uniform across space. The analysis of the number of significant correlations (|ρ| > 0.40) showed that ENTR exhibits more positive associations with $T_{max}$ and $T_{min}$ (exceeding 930 grid points) than with precipitation metrics. This implies that extreme temperatures are more strongly related to general atmospheric instability levels. In contrast, for precipitation—especially $P_{min}$—negative correlations dominated, indicating that low entropy (representing more ordered conditions) is often linked to drought scenarios.

The entropy gradient (|∇ENTR|), reflecting the spatial dynamics of variability, showed fewer positive correlations across all variables, though the number of negative correlations for $P_{min}$ (695) and $P_{max}$ (513) remained high. This supports the interpretation that rapid shifts in atmospheric instability are particularly associated with extreme precipitation events, highlighting the prognostic utility of |∇ENTR| in this context.

The consistency in the signs of correlations between ENTR and |∇ENTR| was relatively low—approximately 45% for all variables—which suggests that the two metrics capture distinct mechanisms underlying extreme events. The mean values of positive correlations for ENTR were highest for temperature variables (around 0.33) and lowest for $P_{min}$ (0.240), implying that elevated entropy values tend to promote thermal extremes more than hydrological ones. Conversely, the most pronounced negative correlations for ENTR were also linked to $P_{min}$ (–0.298), reinforcing the hypothesis that lower entropy favors highly ordered, dry atmospheric states.

The entropy gradient generally showed stronger negative correlations than positive ones, with mean values for $P_{min}$ and $P_{max}$ surpassing −0.3. This emphasizes the sensitivity of precipitation variables to short-term dynamic fluctuations in atmospheric structure. Differences in standard deviations across variables further indicate that temperature correlations are more dispersed, whereas those for $P_{min}$ are more concentrated around strong negative values.

From the perspective of information transfer, ENTR appears to better reflect long-term atmospheric instability associated with thermal extremes. In contrast, |∇ENTR| is more useful for detecting short-term, transitional episodes of extreme precipitation. This underscores the need to differentiate between static (ENTR) and dynamic (|∇ENTR|) informational structures in extreme weather analysis. Such differentiation is especially relevant in the design of early warning systems, which should incorporate both the level of climatic uncertainty (entropy) and the rate at which it changes (entropy gradient).

Ultimately, the presented data suggest that the strongest prognostic potential of ENTR and its gradient lies in their application to thermal extremes and minimum precipitation. These insights carry direct implications for the monitoring and forecasting of heatwaves and droughts.

Table 4 presents a comparative analysis of the maximum seasonal–spatial Spearman correlations between entropy (ENTR) and its temporal derivative (dENTR/dt) with key meteorological variables: maximum temperature, minimum temperature, maximum precipitation, and minimum precipitation. The Kolmogorov–Smirnov test revealed that the correlation distributions between ENTR and dENTR/dt with $T_{max}$ deviated significantly from normality (*p* < 10^−41^), highlighting the strong nonlinearity in the relationship between information flow and extreme temperature values. Although *p*-values for other variables were higher (e.g., *p* = 0.0383 for $P_{max}$), they still indicate statistically significant differences, albeit of lower intensity.

The greatest number of significant correlations (|ρ| > 0.40) was observed between ENTR and $T_{max}$, with 1098 positive and 966 negative correlations. This strongly confirms an informational dependency between entropy and extreme maximum temperatures. These values were substantially higher than those for dENTR/dt, which yielded 486 and 426 positive and negative correlations, respectively. For $T_{min},\;P_{max}\;\;\mathrm{and}\;P_{min}$, the number of significant correlations was more balanced and overall smaller, suggesting these variables possess a lower informational linkage with entropy metrics.

Strikingly, the concordance in correlation signs between  ENTR and dENTR/dt was low—around 27–28% for all variables—which reinforces the idea that static entropy and its dynamics convey different types of information about meteorological extremes. The mean values of positive correlations for ENTR were highest for $T_{max}$ (0.415), suggesting that elevated entropy is directly associated with heatwave occurrences. Conversely, the average of negative correlations reached −0.401, implying that low entropy may also be reflective of mechanisms that suppress temperature extremes.

In contrast, correlations with dENTR/dt were more symmetrical and balanced around ±0.33–0.34, indicating that entropy variability is more aligned with transitional meteorological changes than with absolute values. Moreover, the lower standard deviations of correlations for dENTR/dt suggest that this derivative offers more uniform but generally weaker informational signals than ENTR .

The implications drawn from Table 4 are important for understanding the flow of information in the context of weather extremes. ENTR reflects accumulated atmospheric instability, where elevated levels promote the occurrence of extreme $T_{max}$ values. Meanwhile, its temporal derivative (dENTR/dt) better captures the dynamic changes leading up to extreme events. Particularly notable is that for the precipitation variables ($P_{max}$and $P_{min}$), ENTR and dENTR/dt reached similar mean correlation values. This suggests that the information flow associated with precipitation extremes is not solely a function of instability level but also of its fluctuations over time.

In summary, ENTR and dENTR/dt are complementary informational metrics. ENTR is more effective for assessing long-term trends—especially for extreme temperatures—while dENTR/dt proves more useful in the short-term forecasting of meteorological changes. Such dual-perspective analysis is of significant value for the development of early warning models and for improving predictions of climate extremes.

The regional variation in the strength of correlations between information entropy and extreme values of temperature and precipitation across Europe can be explained by the influence of complex climatic, physical, and orographic mechanisms that shape local atmospheric dynamics.

Shannon entropy, calculated from the joint distribution of temperature and precipitation, measures the level of uncertainty and irregularity of these variables. In Southern and Southeastern Europe—including regions such as Greece, the Balkans, and Turkey—strong positive correlations between entropy and maximum temperature as well as extreme precipitation were observed. This is likely due to the frequent co-occurrence of convective phenomena, thunderstorms, and heatwaves—features typical of Mediterranean and continental climates, where highly seasonal weather conditions prevail. In these regions, entropy effectively captures both the frequency and intensity of atmospheric variability, leading to strong alignment with extreme meteorological indicators.

In Central and Eastern Europe (e.g., Poland, the Czech Republic, Ukraine), positive correlations were also frequently significant, though their spatial distribution was more fragmented. This may reflect the influence of a transitional temperate climate, which is subject both to westerly circulation and to continental advection of warm or cold air masses. The orographic complexity of mountainous regions (e.g., the Alps and Carpathians) further enhances local weather variability, resulting in more dispersed statistical relationships between entropy and extremes—as confirmed by contrasting patterns in correlation analyses. In such areas, high entropy does not always coincide with maximum values of temperature or precipitation, but often reflects microscale atmospheric dynamics, shaped by topography, slope exposure, and local circulation patterns.

In contrast, Northwestern Europe—including the United Kingdom, Scandinavia, and the North Sea region—showed weak or statistically insignificant correlations between entropy and extremes. The maritime climate in these areas is characterized by thermal stability, a relatively uniform precipitation pattern, and frequent stratus cloud cover, all of which suppress both extreme amplitudes and interperiod variability. Under such conditions, entropy exhibits low sensitivity to weather changes, as climate variability is dampened by the influence of the ocean and the presence of frequent low-pressure systems, which dissipate localized anomalies. Additionally, cooler air masses over Scandinavia reduce convective activity, lowering the occurrence of intense precipitation and temperature extremes.

Urban regions and lowland areas with mild climates (e.g., northern France, the Benelux countries) also exhibit relatively homogenized weather conditions, leading to lower entropy values and weaker correlations with extremes. It is also important to note the seasonal dimension of these relationships: analyses indicate that entropy peaks during summer months (particularly June–August) and most frequently correlates with extreme conditions. This results from increased convective activity, storms, and heatwaves. In winter, while entropy values generally decline, in regions experiencing growing instability (e.g., Central Europe), significant correlations with extremes may still emerge, primarily due to more frequent abrupt shifts in synoptic conditions.

Thus, the variability in correlation strength stems from a combination of thermal, circulatory, and local geographic factors. Additionally, the analysis included trend (Mann–Kendall) and change point (Pettitt) tests, which helped identify regions with intensifying entropy trends over time—further influencing their relationship with extreme meteorological parameters. For instance, in areas where entropy shows an upward trend, the potential linkage to extreme weather events—such as droughts or heatwaves—also increases.

Ultimately, the observed spatial and seasonal differences in correlation underscore the necessity of a regionally tailored approach to interpreting entropy as an indicator of atmospheric variability. They also highlight the importance of accounting for local climatic and physical features when forecasting weather-related risks.

## 6. Summary

The applied methodology—integrating statistics, information theory, and the analysis of nonlinear dynamical systems—constitutes an innovative tool for diagnosing climate instability and forecasting extreme weather events. The use of Shannon entropy, derived from the joint distribution of temperature and precipitation, enables a detailed assessment of seasonal and spatial climate variability at high resolution. The model is grounded in a well-established mathematical formalism that incorporates both the gradient and divergence of entropy, allowing for a rigorous analysis of atmospheric processes dispersed in time and space.

Both entropy and its spatial gradient proved to be effective indicators of thermal and hydrological risk. In particular, the entropy gradient (∇H) enabled the detection of spatial weather contrasts and may serve as an early indicator of drought, especially in southern Europe. The relationships between the entropy gradient and meteorological variables were particularly strong for maximum temperature and precipitation in climatically dynamic regions such as the Balkans, Scandinavia, and Central Europe, while correlations with minimum variables were more heterogeneous, reflecting the influence of local factors.

Moreover, the temporal derivative of entropy (∂H/∂t) revealed nonlinear relationships between local weather dynamics and extreme events, exhibiting high Spearman correlation values with moderate time lags. In many regions, its spatial structure aligned with the distribution of climatic zones, further supporting its prognostic utility. The information entropy formalism facilitates the identification of areas particularly susceptible to extreme weather, supports the development of early warning systems, and enables the exploration of spatial–temporal patterns not captured by traditional statistical methods.

The inclusion of derived variables such as the entropy flux vector ($J_{H}$) and its divergence ($\nabla \cdot J_{H}$) allows for the classification of regions as generators or suppressors of atmospheric variability, in accordance with the principles of the Fokker–Planck equations [98]. In this framework, regions with positive divergence are interpreted as active generators of variability, while areas with negative divergence function as stabilizers of the climate system.

This approach, rooted in field theory and continuous physics, can be integrated with existing meteorological and hydrological systems. Its extension to include additional variables—such as humidity, solar radiation, or wind—would enable a more comprehensive representation of the climate system. Overall, the analysis of entropy and its derivatives provides novel, high-quality tools for studying atmospheric variability and climate adaptation, offering a coherent framework for describing the spatiotemporal structure of weather information.

The application of the findings from this study in the context of real-world climate monitoring systems and early warning mechanisms opens new avenues for the development of climate services and decision-support tools. The spatiotemporal analysis of information entropy, integrated with dependency modeling via copulas, enables the identification of areas particularly vulnerable to atmospheric instability and extreme weather events.

By mapping entropy sources and sinks, it becomes possible to detect regions with increasing risk of droughts, heatwaves, or intense rainfall—often before these phenomena are captured by conventional forecasts. Such signals can be successfully incorporated into early warning systems, enhancing their sensitivity to unusual patterns of weather variability.

Moreover, the methods used in this study allow for the quantification of uncertainty and forecast reliability, which is essential for informed decision-making under climate variability. Climate services that integrate this type of information can provide tailored alerts—for example, to support agriculture, water resource management, or energy planning.

The results can also be applied to calibrate climate risk indices and optimize local adaptation strategies. Integrating entropy-based indicators with data from satellites and ground-based monitoring stations enables the real-time tracking of changes and supports the development of adaptive predictive models.

As a result, the proposed methodology can significantly enhance the operational capacity of institutions responsible for climate and economic security. In light of the growing need for precise weather risk management, the use of entropy as a supporting indicator in early warning systems appears particularly promising.

## Figures and Tables

**Table 1:** Analyzed distribution forms for temperature and precipitation variables [55].

Distribution Name	Mathematical Form	Distribution Parameters	No Eq.
Generalized Extreme Value	$f\left(x\;/k,\mu ,\sigma \right)=\left(\frac{1}{\sigma }\right)exp\left(-{\left(1+k\frac{\left(x-\mu \right)}{\sigma }\right)}^{-\frac{1}{k}}\right){\left(1+k\frac{\left(x-\mu \right)}{\sigma }\right)}^{-1-\frac{1}{k}}$ $\mathrm{for}\;\left(1+k\frac{\left(x-\mu \right)}{\sigma }\right)$ >0 and k≠0	μ, σ, k—location parameter, scale parameter and shape parameter	(1)
Normal	$f(x|\mu ,\sigma \;\;)=\frac{1}{\sigma \sqrt{2\pi }}exp\left(-\frac{{\left(x-\mu \right)}^{2}}{2\sigma^{2}}\right)$	σ, μ—standard deviation, and mean	(2)
Lognormal	$f(x|\;\mu ,\sigma \;)=\frac{1}{x\sigma \sqrt{2\pi }}exp\left(\frac{-{\left(logx-\mu \right)}^{2}}{2\sigma^{2}}\right)$ for x>0	σ, μ—distribution parameters	(3)
Weibull	$f(x|a,b\;)=\frac{b}{a}{\left(\frac{x}{a}\right)}^{b-1}exp(-{\left(\frac{x}{a}\right)}^{b})$	a,b —standard deviation, and mean	(4)
Gamma	$f(x|\;a,b\;)=\frac{1}{b^{a}\Gamma (a)}x^{a-1}exp(-\frac{x}{b})$	a,b, Γ(a)—distribution parameters, and the Gamma function	(5)
Extreme Value	$f\left(x|\;\mu ,\sigma \;\;\right)=\sigma^{-1}exp\left(\frac{x-\mu }{\sigma }\right)exp\left(-exp\left(\frac{x-\mu }{\sigma }\right)\right)$	μ, σ, k—location parameter, scale parameter	(6)
Nakagami	$f(x|\;\mu ,\omega \;)=2{\left(\frac{\mu }{\omega }\right)}^{\mu }\frac{1}{\Gamma \left(\mu \right)}x^{2\mu -1}exp(-\frac{\mu }{\omega }x^{2})$ $\;\mathrm{for}\;\;x\geq 0,\;\;\omega >0,\;\;\mu \geq \frac{1}{2}$	μ,ω—shape parameter, scale parameter	(7)

**Table 2:** Analyzed forms of bivariate copula functions.

Copula Functions	Mathematical Form	Kendall’s τ	Eq.
Gaussian	$\frac{1}{\sqrt{1-\rho^{2}}}exp\left(\frac{\left(a^{2}+b^{2}\right)\rho^{2}-2ab\rho }{2\left(1-\rho^{2}\right)}\right)$ where $\rho \in \left(-1,1\right),$ $a=\sqrt{2}{erf}^{-1}\left(2u-1\right)$, $b=\sqrt{2}{erf}^{-1}\left(2v-1\right)$ $\mathrm{erf}\left(z\right)=\frac{2}{\sqrt{\pi }}\int_{0}^{z}exp\left(-t^{2}\right)dt$	$\tau =\frac{2}{\pi }arcsin\left(\theta \right)$	(8)
Clayton	$\;max\left[{\left(u^{-\theta }+v^{-\theta }-1\right)}^{-\frac{1}{\theta }}\right]$ where θ∈[−1,∞)\{0}	$\tau =\frac{\theta }{1+\theta }$	(9)
Frank	$-ln\left(\frac{1+g\left(u\right)g\left(v\right)}{g\left(1\right)}\right)\theta^{-1}\;$ where $g\left(x\right)=exp(-\theta x)-1$ θ∈R\{0}	$\tau =1-\frac{4}{\theta }+\frac{4}{\theta^{2}}\int_{0}^{\theta }\frac{t}{e^{t}-1}dt$	(10)
Gumbel	$exp(-{\left({\left(-lnu\right)}^{\theta }+{\left(-lnv\right)}^{\theta }\right)}^{\frac{1}{\theta }})$ where θ∈[1,∞)	$\tau =\frac{\theta -1}{\theta }$	(11)

**Table 3:** Comparative Analysis of Maximum Temporal Spearman Correlations between ENTR and |∇ENTR| with Meteorological Variables.

Criteria	$T_{max}$	$T_{min}$	$P_{max}$	$P_{min}$
Kolmogorov–Smirnov test	stat = 0.162	stat = 0.163	stat = 0.164	stat = 0.086
	*p* = 1.7 × 10^−43^	*p* = 8.13 × 10^−44^	*p* = 1.01 × 10^−44^	*p* = 2.71 × 10^−12^
Shapiro–Wilk test—Royston’s algorithm (1992)	stat = 0.995	stat = 0.994	stat = 0.985	stat = 0.995
	*p* = 3.19 × 10^−11^/H0 = 1	*p* = 7.07 × 10^−12^/H0 = 1	*p* = 0/H0 = 1	*p* = 0/H0 = 1
Number of correlations |ρ| > 0.40				
ENTR positive	930	931	741	333
ENTR negative	299	295	489	693
|∇ENTR| positive	453	441	306	169
|∇ENTR| negative	555	573	513	695
Concordance of correlation sign (ENTR vs.|∇ENTR|)	44.68%	44.73%	46.03%	46.53%
Positive and negative correlation statistics				
MEAN ENTR positive	0.329	0.33	0.295	0.240
STD ENTR positive	0.222	0.221	0.19	0.171
MEAN ENTR negative	−0.237	−0.245	−0.287	−0.298
STD ENTR negative	0.181	0.183	0.219	0.205
MEAN |∇ENTR| positive	0.295	0.295	0.248	0.219
STD |∇ENTR| positive	0.214	0.214	0.176	0.162
MEAN |∇ENTR| negative	−0.304	−0.311	−0.285	−0.312
STD |∇ENTR| negative	0.211	0.215	0.197	0.205

**Table 4:** Comparative Analysis of Maximum Seasonal-Spatial Spearman Correlations between ENTR and dENTR/dt with Meteorological Variables.

Criteria	$T_{max}$	$T_{min}$	$P_{max}$	$P_{min}$
Kolmogorov–Smirnov test	stat = 0.151	stat = 0.055	stat = 0.031	stat = 0.031
	*p* = 4.93 × 10^−42^	*p* = 7.1 × 10^−6^	*p* = 0.0383	*p* = 0.0318
Shapiro–Wilk test—Royston’s algorithm (1992)	stat = 0.865	stat = 0.861	stat = 0.860	stat = 0.848
	*p* = 0/H0 = 1	*p* = 0/H0 = 1	*p* = 0/H0 = 1	*p* = 0/H0 = 1
Number of correlations |ρ| > 0.40				
ENTR positive	1098	397	500	527
ENTR negative	966	520	518	545
dE/dt positive	486	452	517	475
dE/dt negative	426	477	500	442
Concordance of correlation sign (ENTR vs dENTR)	27.74%	27.72%	28.10%	27.15%
Positive and negative correlation statistics				
MEAN ENTR positive	0.415	0.330	0.341	0.343
STD ENTR positive	0.117	0.093	0.097	0.096
MEAN ENTR negative	−0.401	−0.337	−0.340	−0.344
STD ENTR negative	0.115	0.092	0.092	0.094
MEAN dENTR positive	0.338	0.335	0.338	0.339
STD dENTR positive	0.088	0.084	0.086	0.086
MEAN dENTR negative	−0.334	−0.337	−0.342	−0.333
STD dENTR negative	0.087	0.089	0.089	0.086

## Data Availability

Dataset available on request from the author.
